# Aerobic denitrification as an N_2_O source from microbial communities

**DOI:** 10.1093/ismejo/wrae116

**Published:** 2024-06-24

**Authors:** Nina Roothans, Minke Gabriëls, Thomas Abeel, Martin Pabst, Mark C M van Loosdrecht, Michele Laureni

**Affiliations:** Department of Biotechnology, Delft University of Technology, van der Maasweg 9, 2629 HZ Delft, the Netherlands; Department of Biotechnology, Delft University of Technology, van der Maasweg 9, 2629 HZ Delft, the Netherlands; Delft Bioinformatics Lab, Delft University of Technology, van Mourik Broekmanweg 6, Delft 2628 XE, the Netherlands; Infectious Disease and Microbiome Program, Broad Institute of MIT and Harvard, 415 Main Street, Cambridge, MA 02142, United States; Department of Biotechnology, Delft University of Technology, van der Maasweg 9, 2629 HZ Delft, the Netherlands; Department of Biotechnology, Delft University of Technology, van der Maasweg 9, 2629 HZ Delft, the Netherlands; Department of Biotechnology, Delft University of Technology, van der Maasweg 9, 2629 HZ Delft, the Netherlands; Department of Water Management, Delft University of Technology, Stevinweg 1, 2628 CN Delft, the Netherlands

**Keywords:** aerobic denitrification, nitrous oxide, oxic/anoxic cycling, microbial enrichment

## Abstract

Nitrous oxide (N_2_O) is a potent greenhouse gas of primarily microbial origin. Oxic and anoxic emissions are commonly ascribed to autotrophic nitrification and heterotrophic denitrification, respectively. Beyond this established dichotomy, we quantitatively show that heterotrophic denitrification can significantly contribute to aerobic nitrogen turnover and N_2_O emissions in complex microbiomes exposed to frequent oxic/anoxic transitions. Two planktonic, nitrification-inhibited enrichment cultures were established under continuous organic carbon and nitrate feeding, and cyclic oxygen availability. Over a third of the influent organic substrate was respired with nitrate as electron acceptor at high oxygen concentrations (>6.5 mg/L). N_2_O accounted for up to one-quarter of the nitrate reduced under oxic conditions. The enriched microorganisms maintained a constitutive abundance of denitrifying enzymes due to the oxic/anoxic frequencies exceeding their protein turnover—a common scenario in natural and engineered ecosystems. The aerobic denitrification rates are ascribed primarily to the residual activity of anaerobically synthesised enzymes. From an ecological perspective, the selection of organisms capable of sustaining significant denitrifying activity during aeration shows their competitive advantage over other heterotrophs under varying oxygen availabilities. Ultimately, we propose that the contribution of heterotrophic denitrification to aerobic nitrogen turnover and N_2_O emissions is currently underestimated in dynamic environments.

## Introduction

Nitrous oxide (N_2_O) is today’s third most important greenhouse gas and the main stratospheric ozone-depleting substance [1]. Globally, the majority of N_2_O originates from biological conversions in natural, managed, and engineered ecosystems [2], such as oceans [3], agricultural soils [4], and wastewater treatment plants [5]. N_2_O emissions from anthropogenic activities are projected to reach 11.5 Tg N yr^−1^ in 2050, double the amount emitted in 2000, if no mitigation action is taken [1, 2]. Robust emission control strategies strongly rely on our knowledge of the microbiology underlying N_2_O turnover.

N_2_O is a metabolic by-product of autotrophic nitrification, the aerobic oxidation of ammonium (NH_4_^+^) to nitrite (NO_2_^−^) and nitrate (NO_3_^−^), and an obligate intermediate of heterotrophic denitrification, the multi-step reduction of NO_3_^−^ to dinitrogen gas (N_2_). Conventionally, nitrification and denitrification are considered to dominate N_2_O emissions in the presence and absence of O_2_, respectively [3, 4, 6]. Oxygen is known to regulate the expression and inhibit the activity of denitrifying enzymes [7–9]. Besides, as most known denitrifiers are facultative aerobes, the more energetically and kinetically favourable O_2_ respiration is expected to be prioritised over denitrification in oxic conditions [10]. The aerobic contribution of denitrification is thus generally neglected in soils [11–13], oceans [3, 14], and wastewater treatment systems [15–17]. However, starting from the seminal work of Robertson & Kuenen [18], the occurrence of denitrification under high oxygen concentrations has been documented in pure culture studies (as previously reviewed [10]). What remains to be resolved is the ecological significance of heterotrophic denitrification in aerobic N_2_O formation.

*Sensu stricto*, we refer to the simultaneous occurrence of heterotrophic denitrification and aerobic respiration as aerobic denitrification [18–21]. Biochemically, the co-respiration of O_2_ and nitrogen oxides by the same organism may result from the *de novo* aerobic synthesis of denitrifying enzymes or from the residual activity of anaerobically expressed enzymes [8]. Based on a past literature review [10], aerobic denitrification rates seem to be generally much lower than the anaerobic ones, yet are likely to provide an ecological advantage in dynamic environments. Bacteria reported to denitrify aerobically, including *Alcaligenes faecalis* and multiple *Pseudomonas* species, have indeed been successfully isolated mainly from ecosystems exposed to fluctuating O_2_ levels such as soils, sediments, and activated sludge [18, 21–23]. One study [21] employed weekly alternating oxic/anoxic conditions to enrich for aerobic denitrifiers prior to isolation, further highlighting dynamic O_2_ conditions as key to select for bacteria capable of denitrifying under oxic conditions. However, most reported aerobic heterotrophic denitrification rates are based on a limited number of isolates characterised primarily under continuous aeration, inherently hindering their extrapolation to complex microbiomes in dynamic O_2_ environments. Central challenges in open ecosystems are the co-occurrence of nitrification as a potential confounding aerobic N_2_O source and the development of anoxic micro-niches in microbial aggregates [24, 25]. Only three studies quantified denitrification in the presence of oxygen in natural communities, namely in soil bacteria extracted by density-gradient centrifugation [25] and intact sea sediments [20, 24]. All authors experimentally showed nitrification to be negligible, yet anoxic niches could not be excluded in these complex ecosystems. One study [24] even observed a marked decrease in aerobic NO_x_^−^ respiration upon vigorous stirring, possibly resulting from the disruption of anoxic micro-niches. The extent to which heterotrophic denitrification contributes to overall aerobic nitrogen turnover in dynamic ecosystems is currently unknown.

We enriched for two communities of heterotrophic denitrifiers co-respiring O_2_ and NO_3_^−^ under alternating oxic/anoxic conditions to quantitatively resolve the ecological role of aerobic denitrification. Our underlying hypothesis was that the ability to aerobically respire nitrogen oxides provides a competitive advantage in complex microbiomes exposed to fluctuating oxygen availabilities. We use open culturing techniques that mimic natural ecosystems, allowing microbial communities to evolve under non-axenic conditions, and the fittest organisms for the imposed conditions to dominate. Highly aerated planktonic cultures were employed to exclude anoxic micro-niches, whereas continuous allylthiourea (ATU) addition ensured full suppression of nitrification, eliminating it as possible confounding N_2_O source. The genetic potential and actual metabolism of each community member was characterised by metagenomic and metaproteomic analyses. This study proves the selective advantage of oxygen and nitrogen oxides co-respiration and quantifies its potential contribution to nitrogen turnover and N_2_O emissions in complex communities. Our findings also suggest that the contribution of heterotrophic denitrification to aerobic N_2_O emissions may currently be underestimated.

## Materials and methods

### Continuous-flow stirred tank reactors operation

Two 1-L jacketed continuous-flow stirred tank reactors (Applikon, Getinge) were operated during 96 days, with continuous and vigorous mixing at 500 rpm using a six-blade turbine. The hydraulic and sludge retention times (HRT and SRT) were identical, controlled at 2 ± 0.1 days by two peristaltic pumps (Masterflex) continuously feeding the two media to the system and an effluent pump removing 94 ml of broth every 6 h. The average working volume was 0.75 ± 0.05 L. The temperature was controlled at 20 ± 0.1°C using a cryostat bath (Lauda). The pH and dissolved oxygen were continuously monitored by pH and dissolved oxygen probes (Applikon AppliSens, Getinge). The pH was kept at 7.1 ± 0.1 by 1-M HCl or 1-M NaOH with two peristaltic pumps (Watson Marlow) controlled by a process controller (Applikon in-Control, Getinge).

Denitrifying bacteria were enriched by continuous supply of 0.93 ± 0.04 N-mmol/h NO_3_^−^ as electron acceptor and a mixture of volatile fatty acids (VFAs) as electron donor and carbon source: acetate (0.94 ± 0.08 C-mmol/h), propionate (1.00 ± 0.09 C-mmol/h), and butyrate (0.75 ± 0.07 C-mmol/h). Ammonia served as the nitrogen source. The reactors were covered with aluminium foil to prevent the growth of phototrophic organisms. Nitrogen and carbon media were prepared separately to prevent microbial growth during storage. Nitrogen medium consisted of (per litre): 9.14-g NaNO_3_, 2.84-g NH_4_Cl, 2.01-g KH_2_PO_4_, 1.04-g MgSO_4_ · 7 H_2_O, 0.04-g NaOH, 4-mg yeast extract, 5-ml trace element solution [26], and 1-ml of a 10 g/L solution of allylthiourea (ATU). ATU was added to selectively inhibit bacterial ammonium oxidation to nitrite [27–29] without significantly affecting denitrification [30]. The trace element solution consisted of (per litre): 50-g EDTA · H_2_ · Na_2_ · 2 H_2_O, 2.5-g FeSO_4_ · 7 H_2_O, 1.1-g ZnSO_4_ · 7 H_2_O, 4.1-g CaCl_2_ · 2 H_2_O, 2.2-g MnSO_4_ · H_2_O, 1.1-g Na_2_MoO_4_ · 2 H_2_O, 0.8-g CuSO_4_ · 5 H_2_O, and 0.7-g CoCl_2_ · 6 H_2_O. Carbon medium consisted of (per litre): 8.1-g NaCH_3_OO · 3 H_2_O, 1.9-ml C_4_H_8_O_2_, and 4.1-g NaC_3_H_5_O_2,_ the pH was set to 6.0 with NaOH pellets. After the initial start-up phase of 27 days, the VFAs were always below detection limit in the effluent, confirming carbon-limiting conditions. Drops of antifoam C emulsion (Merck Life Science NV), diluted six times, were added to the reactors when foam formation was noted.

The reactors were inoculated with activated sludge from the Amsterdam-West wastewater treatment plant, comprising 349 high-quality metagenome-assembled genomes (MAGs), 305 of which had at least one denitrification gene (genome-resolved metagenomic composition in Supplementary Fig. S20) [31]. Carbon-limiting conditions were reached after an initial start-up phase of 20 days where NO_3_^−^ was the growth-limiting compound. During the NO_3_-limiting start-up phase, the concentrations of VFAs were increased by four times in the carbon medium compared to the values presented above. The two reactors were exposed to continuous cycles of alternating oxic and anoxic conditions in a time proportion of 2:1. The reactors were exposed to 4 (R_4_) or 32 (R_32_) cycles per day, with oxic periods of 4 h and 30 min and anoxic periods of 2 h and 15 min, respectively. Oxic and anoxic conditions were maintained by continuous sparging of compressed air and N_2_, respectively, at 400 ml/min, controlled by mass-flow controllers (Brooks). Oxic conditions close to air saturation were assured by maintaining average dissolved O_2_ concentrations of 7.5 ± 0.2 and 6.8 ± 0.3 mg/L in R_4_ and R_32_, respectively. The reactors reached fully anoxic or oxic conditions within 5 min after switching the influent gas. The 6-h reactor broth removal coincided with the end of an anoxic phase. The net amount of oxic (≥1% air saturation) and anoxic (<1% air saturation) hours per day were 16:8 and 17:7 for R_4_ and R_32_, respectively. Throughout the operation, visual and microscopic analysis confirmed that the cultures remained planktonic and homogeneous (Supplementary Fig. S4). For R_32_, small biomass aggregates were progressively washed out reaching an entirely homogeneous and suspended culture after 63 days of operation. Occasional biomass accumulation in the splash zone of the bioreactor was always removed with no noticeable consequential changes in the reactors’ operation (Supplementary Fig. S2), confirming that it played no role in the nitrogen conversions. The measured O_2_ conversion rates were 7.5-fold lower than the maximum O_2_ transfer rate (Supplementary Table S1 and Equation S9), reflecting the significant aeration overcapacity in the reactors.

For metabolite and biomass analysis, quadruplicate samples of 2 ml were taken from both reactors at three moments within a cycle: at the start and end of the oxic phase, and at the end of the anoxic phase. The samples were placed on ice and immediately filtered using 0.22-μm PVDF Millex-GV syringe filters (Merck) or centrifuged at 16 200 × *g* for 5 min at 4°C to separate the biomass from the supernatant. The pellets were stored at −80°C and the supernatant at −20°C until further analysis. Feed substrate concentrations were confirmed by occasionally sampling the reactor influent, with storage at −20°C until analysis.

### Analytical methods

The concentrations of NH_4_^+^, NO_2_^−^, and NO_3_^−^ in the influent and effluent supernatant were spectrophotometrically measured with the Gallery Discrete Analyzer (Thermo Fisher Scientific) or cuvette test kits (Hach Lange) immediately after sampling or within 24 h after storage at 4°C. The concentrations of acetate, propionate, and butyrate in the influent and effluent supernatant were measured after storage at −20°C by high-pressure liquid chromatography (Vanquish Core HPLC, Thermo Fisher Scientific) using an Aminex HPX-87H column (300 × 7.8 mm) (Bio-Rad), calibrated with solutions ranging from 0 to 250 mM. The concentrations of O_2_, N_2_O, and CO_2_ in the off-gas were continuously monitored online (every minute) by a Rosemount NGA 2000 off-gas analyser (Emerson). Before reaching the analyser, the off-gas was dried in a condenser, operated with water at 4°C using a cryostat bath (Lauda).

### Calculations

The calculations of consumption and production rates of all compounds are detailed in Supplementary Section 2. Briefly, the overall consumption and production rates of dissolved compounds (R_i_, with i = NH_4_^+^, NO_2_^−^, NO_3_^−^, acetate, propionate, and butyrate) were calculated via a mass balance of the volumetric influent and effluent (F_i,in_ and F_i,out_) flow rates, and the influent and effluent concentrations (C_i,in_ and C_i,out_) measured in triplicate:


(1) \begin{equation*} {\mathrm{R}}_{\mathrm{i}}^{\mathrm{overall}}={\mathrm{F}}_{\mathrm{i},\mathrm{out}}\cdot{\mathrm{C}}_{\mathrm{i},\mathrm{out}}-{\mathrm{F}}_{\mathrm{i},\mathrm{in}}\cdot{\mathrm{C}}_{\mathrm{i},\mathrm{in}} \end{equation*}

The overall rates (R_i_^overall^) are, in practice, a weighted average of the aerobic and anaerobic consumption and production rates (R_i_^aerobic^ and R_i_^anaerobic^), so these three rates are related according to the following equation:


(2) \begin{equation*} {\mathrm{R}}_{\mathrm{i}}^{\mathrm{overall}}=\frac{{\mathrm{t}}_{\mathrm{aerobic}}}{24}\cdot{\mathrm{R}}_{\mathrm{i}}^{\mathrm{aerobic}}+\frac{{\mathrm{t}}_{\mathrm{anaerobic}}}{24}\cdot{\mathrm{R}}_{\mathrm{i}}^{\mathrm{anaerobic}} \end{equation*}

The biomass (X) production rate was estimated from the ammonium consumption rates, assuming complete assimilation into biomass at a ratio of 0.2 N-mol/C-mol. The same estimation was obtained when calculating the biomass rates from the carbon balance (i.e. from the CO_2_ and organic carbon rates), validating the previous assumption. The estimated biomass concentrations were 1.8 ± 0.2 (R_4_) and 2.1 ± 0.3 (R_32_) g·L^−1^. The overall, oxic, and anoxic accumulation rates of gaseous compounds (R_gas,i_, i = N_2_O and CO_2_) were calculated from continuous measurements of the molar fractions in the gas inlet and outlet (y_i,in_ and y_i,out_), the atmospheric pressure (P_atm_), the volumetric gas flow (F_V,gas_), the ideal gas constant (R), and the reactor temperature (T).


(3) \begin{equation*} {\mathrm{R}}_{\mathrm{gas},\mathrm{i}}=\left({\mathrm{y}}_{\mathrm{i},\mathrm{out}}-{\mathrm{y}}_{\mathrm{i},\mathrm{i}\mathrm{n}}\right)\cdot \frac{{\mathrm{P}}_{\mathrm{atm}}\cdot{\mathrm{F}}_{\mathrm{V},\mathrm{gas}}}{\mathrm{R}\cdot \mathrm{T}} \end{equation*}

The overall N_2_ production rate was estimated from the nitrate and N_2_O rates, as the accumulation of nitrite and nitric oxide was negligible throughout steady state. The O_2_ consumption rates (R_O2_) during the oxic phase were calculated from the experimentally determined volumetric mass transfer coefficient (k_L_a, Supplementary Section 1), the O_2_ Henry coefficient (H_O2_), the atmospheric pressure (P_atm_), the O_2_ molar fraction in the off-gas (y_O2_), the continuous dissolved oxygen measurements (DO), and the average broth volume (V).


(4) \begin{equation*} {\mathrm{R}}_{\mathrm{O}2}={\mathrm{k}}_{\mathrm{L}}\mathrm{a}\cdot{\mathrm{H}}_{{\mathrm{O}}_2}\cdot{\mathrm{P}}_{\mathrm{atm}}\cdot{\mathrm{y}}_{{\mathrm{O}}_2}\cdot \left(1-\mathrm{DO}\right)\cdot \mathrm{V} \end{equation*}

For consistency, an ‘overall’ consumption rate was also calculated for O_2_, by averaging its aerobic consumption over the entire cycle duration [Equation (2)]. For all compounds, steady-state rates were determined by averaging the rates measured during the entire steady-state period. Overall carbon and electron balances were calculated from the consumption and production rates of all substrates (R_in_) and products (R_out_), and electron donors (R_eD_) and acceptors (R_eA_), respectively.


(5) \begin{equation*} \mathrm{C}\ \mathrm{balance}\ \left(\%\right)=\frac{{\mathrm{R}}_{\mathrm{in}}}{{\mathrm{R}}_{\mathrm{out}}}=\frac{\left|{\mathrm{R}}_{\mathrm{Ace}}+{\mathrm{R}}_{\mathrm{Pro}}+{\mathrm{R}}_{\mathrm{But}}\right|}{{\mathrm{R}}_{\mathrm{X}}+{\mathrm{R}}_{{\mathrm{CO}}_2}} \end{equation*}

(6) \begin{align*} &{\mathrm{e}}^{-}\ \mathrm{balance}\left(\%\right) =\frac{{\mathrm{R}}_{\mathrm{e}\mathrm{D}}}{{\mathrm{R}}_{\mathrm{e}\mathrm{A}}}=\frac{\left|4\cdot{\mathrm{R}}_{\mathrm{Ace}}+4.7\cdot{\mathrm{R}}_{\mathrm{Pro}}+5\cdot{\mathrm{R}}_{\mathrm{But}}\right|}{-8\cdot{\mathrm{R}}_{{\mathrm{N}\mathrm{O}}_3^{-}}\!-4\cdot{\mathrm{R}}_{{\mathrm{N}}_2\mathrm{O}}\!-3\cdot{\mathrm{R}}_{{\mathrm{N}}_2}\!-4\cdot{\mathrm{R}}_{{\mathrm{O}}_2}\!+4.2\cdot{\mathrm{R}}_{\mathrm{X}}} \end{align*}

The specific aerobic and anaerobic NO_3_^−^ consumption rates were estimated from the available measurements, mass balances, and Equation (2), as explained in Supplementary Section 2. These values were validated with direct calculations from measured concentration profiles throughout each phase (Supplementary Figs S8 and S9). Possible deviations in the estimated rates due to potential PHA accumulation were negligible (Supplementary Section 2).

### DNA extraction, library preparation, and sequencing

DNA was extracted from biomass samples taken at the end of the anoxic period after 68 days of operation using the DNeasy PowerSoil Pro Kit (Qiagen), according to the manufacturer’s instructions with the following exceptions. The pelleted biomass, stored at −80°C, was resuspended in 800 μl of solution CD1 by vortexing before transferring to the PowerBead tube. Samples were homogenised by 4 × 40s bead-beating using the Beadbeater-24 (Biospec) alternated with 2-min incubation on ice. Tubes were gently inverted 10× instead of vortexing to avoid DNA shearing. Elution of the extracted DNA was performed with 50-μl solution C6. The DNA concentration was 710 and 605 ng/μl for R_4_ and R_32_, respectively, as measured with the Qubit 4 Fluorometer (Thermo Fisher Scientific). DNA quality was assessed with the BioTek Synergy HTX multi-mode microplate reader (Agilent). For differential coverage binning and increased bin recovery, DNA was also extracted from samples taken after 41 days of operation using the Dneasy UltraClean Microbial Kit (Qiagen), following the manufacturer’s instructions. The extraction yielded 224 and 267 ng/μl for R_4_ and R_32_, respectively.

Library preparation of the extracted DNA from day 68 for long-read sequencing was performed using the Ligation Sequencing Kit V14 (Oxford Nanopore Technologies Ltd). The NEBNext Companion Module for Oxford Nanopore Technologies Ligation Sequencing (New England BioLabs Inc.) and UltraPure BSA (50 mg/ml) (Thermo Fisher Scientific) were additionally used for the DNA repair and end-prep, and the flow cell priming steps. All steps were performed as instructed by the manufacturer, except the incubations in the Hula mixer were replaced with slow manual inversions (~5 s per inversion). All resuspension steps were performed by flicking the tube. MinION R10.4 version flow cells (Oxford Nanopore), starting with 1345 and 461 active pores, were loaded with 132 and 150-ng DNA for R_4_ and R_32_, respectively. Samples were sequenced in accurate mode (260 bps) for 46 and 40 h, respectively, yielding 14.7 and 4.3 Gbp of sequenced data. Samples from day 41 were sequenced on a NovaSeq 6000 platform (Illumina) by Novogene Ltd. (UK). Approximately 10 Gbp of 150-bp paired-end reads with an insert size of 350 bp were generated.

### Metagenomic data processing

The raw Nanopore data were basecalled using Guppy v6.4.2 (Oxford Nanopore) with the configuration file “dna_r10.4.1_e8.2_260bps_sup.cfg” and --do_read_splitting option. Duplex reads were identified and filtered using the pairs_from_summary and filter_pairs settings from Duplex tools v0.2.19 (Oxford Nanopore), and basecalled with the duplex basecaller of Guppy, using identical settings to the simplex basecalling. The simplex reads, not part of a pair, were merged with the duplex basecalled reads using SeqKit v2.3.0 [32], generating a single fastq file containing all unique reads. Sequences belonging to the Lambda control DNA were removed with NanoLyse v1.2.1 [33]. The basecalled data were inspected and processed with NanoPlot v1.41.0 [33], NanoFilt -q 10 -l 1000 (v2.8.0 [33]), and Porechop v0.2.4 (https://github.com/rrwick/Porechop). Reads assembly was performed with Flye v2.9.1 [34] in --meta mode. Assembly quality was assessed with MetaQUAST v5.0.2 [35] using the --fragmented option. Reads were aligned to the assembly with Minimap2 v2.24 [36]. The assembly was polished with Racon v1.4.3 (https://github.com/isovic/racon) and two rounds of Medaka v1.5.0 (https://github.com/nanoporetech/medaka) with default settings. Nanopore and Illumina reads were mapped to the final assembly using Minimap2 [36], the alignments were converted from SAM to BAM and sorted with SAMtools v1.10 [37], and the contig coverage was calculated with jgi_summarize_bam_contig_depths [38]. Automatic differential coverage binning was independently performed with MetaBAT2 v2.15 [38], MaxBin2 v2.2.7 [39], and CONCOCT v1.1.0 [40], with a minimum contig length of 2000 bp. The output of all binning tools was combined with DAS Tool v1.1.3 [41], using Prodigal v2.6.3 [42] and DIAMOND v2.0.8 [43] for single copy gene prediction and identification, resulting in an optimised non-redundant set of bins. Bin completeness and contamination was determined with CheckM v1.1.3 [44] using the lineage_wf workflow. Nanopore and Illumina bins from each reactor were dereplicated with dRep v3.2.2 [45] with the options -comp 70 -con 10 --S_algorithm gANI, using the default thresholds for average nucleotide identity (ANI). The final set of non-redundant bins (completeness above 70% and contamination under 10%) contained all Nanopore bins and the Illumina bins that did not cluster with any Nanopore bins (gANI <99%). The bins were taxonomically classified with the classify_wf workflow of GTDB-Tk v.2.2.5 [46] using the GTDB release 207 (gtdbtk_r207_v2_data.tar.gz [47]). The relative abundance of each bin in the metagenome was determined with CoverM v0.6.1 (https://github.com/wwood/CoverM) in relative_abundance mode.

Genes were predicted from the assembly using Prodigal v2.6.3 [42] and functionally annotated with DRAM v1.3 in annotate_genes mode [48], using the default settings and the KOfam [49], MEROPS [50], Pfam [51], dbCAN [52], and VOGDB (https://vogdb.org/) databases. Genes of interest were identified by their KO identifier (Supplementary Tables S9–S11). The genes encoding the alpha and beta subunits of the respiratory nitrate reductase (Nar) have the same KO identifiers as the alpha and beta subunits of the nitrite oxidoreductase (Nxr). We could confidently attribute all genes identified with K00370 and K00371 to the nitrate reductase (encoded by *narGHI* or *narZYV*), as the gamma subunit of this enzyme (K00374, exclusive to Nar) was present in all bins containing the alpha and beta subunits. Distinction between clade I and clade II N_2_O reductase (NosZ) was determined by, respectively, identifying the twin-arginine translocation (Tat, IPR006311) or the general secretory (Sec, IPR026468) pathway-specific signal peptides on InterPro v92.0 [53]. The quinol-dependent nitric oxide reductase (qNor, encoded by *norZ*) has a fused quinol oxidase domain on the N-terminal [54], unlike the cytochrome c-dependent reductase (cNor, encoded by *norBC*). Yet, the *norZ* genes were annotated as *norB*, so qNor was distinguished by identifying the quinol oxidase domain through a multiple sequence alignment (COBALT [55]) of putative NorB protein sequences (K04561) with reference sequences of NorB (*Pseudomonas stutzeri*, P98008) and NorZ (*Cupriavidus necator*, Q0JYR9), extracted from UniProtKB [56].

Quality control of the Illumina paired-end reads was performed with FastQC v0.11.7 (https://www.bioinformatics.babraham.ac.uk/projects/fastqc/). Reads were filtered and trimmed with Trimmomatic v0.39 [57] using the options LEADING:3 TRAILING:3 SLIDINGWINDOW:4:15 MINLEN:35 HEADCROP:5. Reads were assembled into contigs using metaspades.py from SPAdes v3.14.1 [58]. The assembly was inspected with MetaQUAST v5.0.2 [35] using the --fragmented option. Contigs smaller than 500 bp were removed with filterContigByLength.pl [59]. Gene prediction and functional annotation was performed identically to the Nanopore data. The paired-end reads were mapped to the contigs using BWA-MEM2 v2.1 [60]. The paired-end reads were mapped to the contigs using BWA-MEM2 v2.1 [60] and the alignments were processed as described above. Automatic binning and bin analysis was identical as described for the Nanopore data, except no differential coverage binning was performed and the default minimum contig length of each binning software was used. The generated bins were further dereplicated with the Nanopore bins as described above. Nonpareil v3.401 [61], ran with the kmer algorithm, estimated that the Illumina reads covered 98.6% and 99.2% of the sample diversity.

### Protein extraction, precipitation, digestion, and clean-up

Preparation of protein samples was performed as previously described [62]. Briefly, biomass samples were homogenised with glass beads (150–212 μm, Sigma Aldrich), 50-mM TEAB buffer with 1% (w/w) NaDOC, and B-PER reagent (Thermo Scientific) through three cycles of vortexing and ice incubation. The samples were incubated at 80°C and sonicated. The supernatant was collected after centrifuging at 14 000 *g*. Proteins were precipitated with 1:4 trichloroacetic acid solution (TCA, Sigma Aldrich) and washed with acetone. The pellet was re-dissolved in 6-M urea (Sigma Aldrich) in 200-mM ammonium bicarbonate, reduced in 10-mM dithiothreitol (Sigma Aldrich) at 37°C for 60 min, and alkylated with 20-mM iodoacetamide (Sigma Aldrich) in the dark for 30 min, at room temperature. Samples were diluted to reach a urea concentration under 1 M. Proteins were digested overnight (21 h) at 37°C with 0.1 μg/μl trypsin (sequencing grade, Promega) dissolved in 1-mM HCl. Samples were desalted and cleaned through solid-phase extraction using an Oasis HLB 96-well μElution Plate (2 mg sorbent per well, 30 μm, Waters) and a vacuum pump. The columns were conditioned with MeOH, equilibrated with two rounds of water, loaded with the digested samples, and washed with two rounds of 5% MeOH. Peptide samples were sequentially eluted with 2% formic acid in 80% MeOH and 1-mM ammonium bicarbonate in 80% MeOH, dried at 50°C in an Integrated SpeedVac System (Thermo Scientific), and stored at −20°C until shotgun proteomic analysis.

### Shotgun metaproteomics

Briefly, samples were dissolved in 20 μl of 3% acetonitrile and 0.01% trifluoroacetic acid. The samples were incubated at room temperature for 30 min and vortexed thoroughly. The protein concentration was measured on a NanoDrop ND-1000 spectrophotometer (Thermo Scientific) at 280-nm wavelength. If needed, samples were diluted to a concentration of 0.5 mg/ml.

Shotgun metaproteomics experiments were performed as recently described [62, 63]. Briefly, aliquots corresponding to ~0.5-μg protein digest were analysed using a nano-liquid-chromatography system consisting of an EASY nano-LC 1200, equipped with an Acclaim PepMap RSLC RP C18 separation column (50 μm × 150 mm, 2 μm, Cat. No. 164568), and a QE plus Orbitrap mass spectrometer (Thermo Fisher Scientific). The flow rate was maintained at 350 nl/min over a linear gradient from 5% to 25% solvent B over 90 min, then from 25% to 55% over 60 min, followed by back equilibration to starting conditions. Solvent A was H_2_O containing 0.1% formic acid (FA), and solvent B consisted of 80% ACN in H_2_O and 0.1% FA. The Orbitrap was operated in data-dependent acquisition (DDA) mode acquiring peptide signals from 385 to 1250 m/z at 70 K resolution in full MS mode with a maximum ion injection time (IT) of 75 ms and an automatic gain control (AGC) target of 3E6. The top 10 precursors were selected for MS/MS analysis and subjected to fragmentation using higher-energy collisional dissociation (HCD) at a normalised collision energy of 28. MS/MS scans were acquired at 17.5-K resolution with AGC target of 2E5 and IT of 75 ms, 1.2 m/z isolation width. Raw mass spectrometric data from each reactor were analysed against a protein reference sequence database respectively constructed from the metagenomic data, including the all MAGs and unbinned portion of the samples taken at day 68 and the additional dereplicated MAGs from day 41, using PEAKS Studio X (Bioinformatics Solutions Inc.) allowing for 20-ppm parent ion and 0.02-m/z fragment ion mass error, three missed cleavages, and iodoacetamide as fixed, and methionine oxidation and N/Q deamidation as variable modifications. Peptide spectrum matches were filtered against 1% false discovery rates (FDRs) and protein identifications with ≥2 unique peptide sequences.

For each protein, the peptide spectral counts were normalised by dividing them with the protein molecular weight. The relative abundance of each protein in the samples was calculated by dividing its normalised spectral counts by the sum of normalised spectral counts of all proteins of that respective sample. The technical duplicates were then averaged. The total relative contribution of each bin to the proteome was determined by summing the relative abundances of its proteins. Similarly, the total relative abundance of functionally identical proteins was determined by summing the relative contribution of all proteins with the same functional annotation. The exclusion of any NapA and NapB peptides in the proteomic data was concluded from the absence of corresponding sequences within the obtained peptide spectrum matches. RStudio v22.0.3 [64] with R v4.2.2 [65], with the plyr v1.8.8 [66], tidyverse v2.0.0 [67], readxl v1.4.2 [68], and ggplot2 v3.4.2 [69] packages, was used for data processing and visualisation.

## Results

### Stable denitrifying cultures under alternating oxygen availability

Two planktonic denitrifying microbial communities were enriched under alternating anoxic and fully oxic conditions to quantitatively resolve the role of aerobic heterotrophic denitrification, i.e. the co-respiration of nitrogen oxides and oxygen, in mixed communities. A mixture of volatile fatty acids (acetate, propionate, butyrate) served as carbon and energy source and NO_3_^−^ as electron acceptor. All dissolved substrates were continuously provided (Supplementary Table S1). The O_2_ supply was controlled to ensure a 1:2 ratio of anoxic to oxic time, split in 4 (R_4_) and 32 (R_32_) cycles per day. Fully anoxic conditions were ensured by continuous N_2_ sparging. In the oxic phase, dissolved oxygen was maintained above 6.5 mg/L (>75% air saturation), and both NO_3_^−^ and O_2_ served as electron acceptors. Continuous supply of allylthiourea (ATU) ensured complete suppression of nitrification, as confirmed by the absence of ammonium oxidation activity (Day 61, Supplementary Fig. S10) and nitrification genes in the recovered metagenomes (Fig. 3).

**Figure 1:**
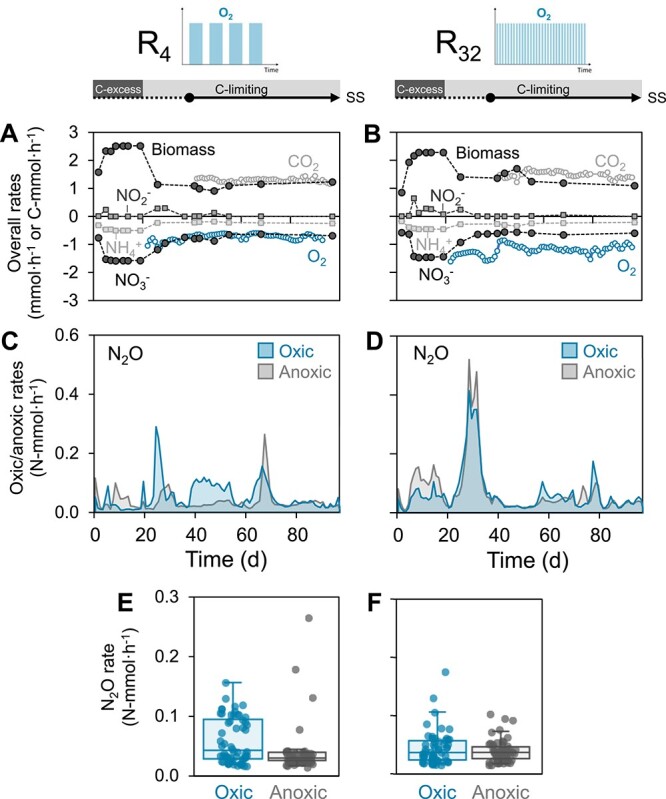
Conversion rates (mmol·h^−1^) in the low- (R_4_) and high-frequency (R_32_) oxic/anoxic cycling reactors over the entire operational period. Prior to the target carbon limiting conditions, the reactors were started up for 20 days under carbon excess. The steady state (SS) was reached on day 37 and maintained for over 2 months (equivalent to 30 generation times). Negative rates represent consumption whereas positive rates represent production. (A, B) Overall (i.e. combined aerobic and anaerobic) NO_3_^−^, NH_4_^+^, and O_2_ consumption, and NO_2_^−^, CO_2_, and biomass production rates (in mmol·h^−1^ or C-mmol·h^−1^ for the carbon compounds). The latter was calculated from the NH_4_^+^ consumption rate. For consistency, an ‘overall’ O_2_ consumption rate was calculated by averaging its aerobic consumption over the entire cycle duration. Error bars of all rates are smaller than the symbols and represent the standard deviation of triplicate samples (nitrogen substrates) or of daily averages of continuous measurements (CO_2_ and O_2_). (C, D) Daily average N_2_O production rates (N-mmol/h) during the oxic and anoxic phases. (E, F) Boxplots summarising the daily N_2_O emission rates (N-mmol/h) in both phases during the SS period.

After a start-up period of 20 days, the reactors were run for 76 days (equivalent to 38 volume changes) under carbon-limiting conditions with a dilution rate of 0.02 h^−1^. The operational steady-state was reached after Day 37, as confirmed by constant overall substrates and products conversion rates (Fig. 1A and B). These overall rates represent the weighted average of the aerobic and anaerobic rates within one cycle [Equation (2)]. For consistency, an ‘overall’ consumption rate was also calculated for O_2_, by averaging its aerobic consumption over the entire cycle duration (Supplementary Section 2). The overall NH_4_^+^, CO_2_, organic carbon, and biomass conversion rates (Supplementary Table S1), as well as the resulting stoichiometric yields (Table 1), were comparable between the two reactors. The enrichments differed only in terms of the overall NO_3_^−^ (Y_NO3/S_) and O_2_ (Y_O2/S_) yields (Table 1). Over the combined oxic and anoxic periods, 56 ± 4% and 39 ± 4% of the total catabolic electron flow was used for NO_3_^−^ reduction in R_4_ and R_32_, respectively, with the remaining being used for O_2_ reduction (Supplementary Table S3). NO accumulation was absent and NO_2_^−^ accumulation was negligible (4 ± 6% and 2 ± 3% of the total consumed NO_3_^−^ for R_4_ and R_32_, respectively, Supplementary Tables S1 and S2) during the entire steady-state period. The carbon and electrons balances closed, further confirming that all involved substrates and products were measured, and supporting N_2_O and N_2_ as the primary products of NO_3_^−^ reduction (Table 1).

**Table 1:** Average overall steady-state stoichiometric yields, and carbon and electron balances in the low- (R_4_) and high-frequency (R_32_) reactors. The yields were calculated using the overall consumption and production rates [i.e. weighted average of the aerobic and anaerobic rates, Equation (2)]. X and S represent biomass and organic substrate, respectively. The standard deviations were calculated from the standard deviation of the consumption and production rates (Supplementary Table S1) using linear error propagation (Equation S3 in supplementary information).

Reactor	Y_N2O/NO3-_ (Nmol/Nmol)	Y_X/S_ (Cmol/Cmol)	Y_NO3-/S_ (Nmol/Cmol)	Y_O2/S_^a^ (mol/Cmol)	Y_CO2/S_ (Cmol/Cmol)	C bal (%)	e^−^ bal (%)
R_4_	0.07 ± 0.04	0.44 ± 0.04	0.30 ± 0.04	0.29 ± 0.03	0.53 ± 0.03	103 ± 5	101 ± 8
R_32_	0.07 ± 0.04	0.46 ± 0.06	0.20 ± 0.01	0.40 ± 0.05	0.51 ± 0.05	103 ± 8	100 ± 8

a For consistency, the O_2_ respiration yield was calculated using the ‘overall’ (i.e. combined aerobic and anaerobic) instead of the aerobic rates.

### Comparable average oxic and anoxic N_2_O production rates

**Figure 2:**
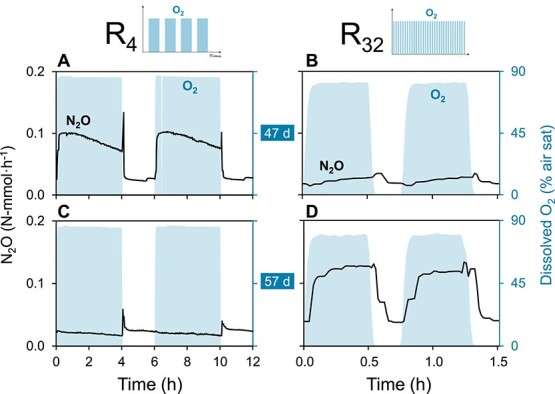
Representative N_2_O profiles during oxic/anoxic periods at steady-state after 47 and 57 days of operation. Left axes: N_2_O accumulation rates in N-mmol·h^−1^ (lines). Right axes: dissolved oxygen concentrations (shaded area). (A, C) Low-frequency reactor (R_4_). (B, D) High-frequency reactor (R_32_).

N_2_O emission by the enrichments was measured throughout the oxic and anoxic phases to assess the ecological significance of denitrification in aerobic N_2_O formation. The aerobic and anaerobic N_2_O production rates remained highly variable throughout the entire operation (Fig. 1C and D, with standard deviations in Supplementary Fig. S1), despite both systems being at operational steady-state (after Day 37), defined by constant conversion rates of the other metabolites. The daily average N_2_O emission rates fluctuated between 0.02 and 0.16 N-mmol·h^−1^ in the two systems. The average N_2_O production rate in R_4_ was higher in the oxic than in the anoxic phase (0.057 ± 0.037 vs. 0.037 ± 0.039 N-mmol/h), whereas these were nearly identical in R_32_ (0.042 ± 0.029 vs. 0.038 ± 0.019 N-mmol/h) (Fig. 1E and F). Throughout the oxic/anoxic cycles, oxic N_2_O accumulation was higher or, at most, equal to the accumulation during anoxia (Fig. 2).

**Figure 3:**
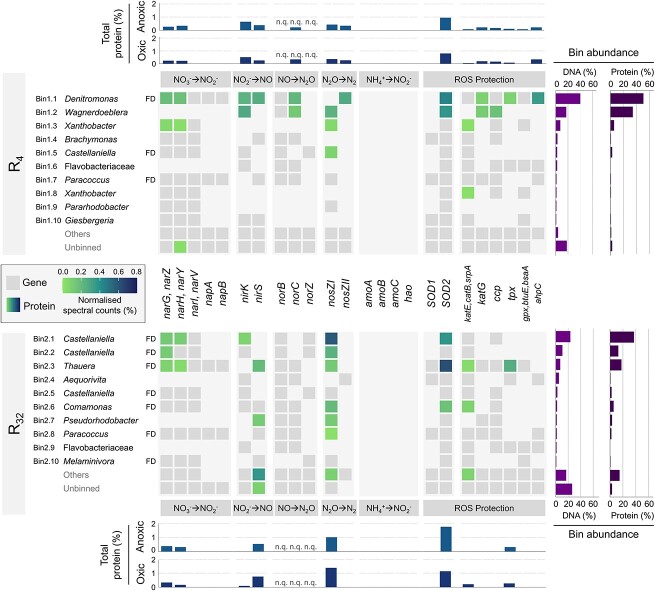
Genomic and proteomic profiles of the top 10 most abundant high-quality MAGs in both enrichments. Gene presence and protein expression in high-quality MAGs (completeness >90% and contamination <5%) in the low- (R_4_) and high-frequency (R_32_) reactors (top panel—R_4_ and lower panel—R_32_), with their respective taxonomic classification at genus (or family if unclassified genus) level. Full denitrifying organisms, with genes encoding for all denitrification steps, are highlighted (FD). Low-abundant high-quality and all medium-quality MAGs (<90% completeness and >5% contamination) were grouped into ‘others’ and low-quality bins (<70% completeness and >10% contamination) were grouped with the unbinned fraction. The presence of genes (grey tiles) and the abundance of their corresponding protein under oxic conditions (coloured tiles) are represented for denitrification (NO_3_^−^ ➔ N_2_), nitrification (NH_4_^+^ ➔ NO_2_^−^), and protection against reactive oxygen species (ROS). The abundance of each protein was determined from peptide spectral sequence counts. Right bar charts: total relative abundance of each MAG in the metagenome (based on relative reads alignment normalised to the corresponding MAG length) and the metaproteome (summed relative abundance of normalised spectral counts of peptides matching to predicted proteins in each MAG). Top/bottom bar charts: total relative abundance of each protein in the oxic and anoxic phases (summed relative abundance of normalised spectral counts); not quantifiable (n.q.): the used methods are not optimised for membrane proteins such as the nitric oxide reductase.

The high aerobic N_2_O production implies that denitrification was active at fully oxic conditions (>6.5 mg O_2_/L). The aerobic and anaerobic NO_3_^−^ consumption rates were estimated based on the aerobic and anaerobic organic substrate and oxygen consumption, CO_2_ production and N_2_O accumulation rates, and the electron balances in each phase (Supplementary Section 2). The estimated aerobic NO_3_^−^ consumption rates were only 2.4- and 7.7-fold lower than the anaerobic rates in R_4_ and R_32_, respectively. This is equivalent to 36 ± 7% and 11 ± 11% of the total aerobic electron flow in each reactor. These values were validated with direct calculations from measured concentration profiles throughout each phase (Supplementary Figs S8 and S9). The fraction of NO_3_^−^ emitted as N_2_O during aeration was estimated to be 12 ± 8% (R_4_) and 24 ± 29% (R_32_).

### Denitrifiers-enriched microbial communities

A metagenomic analysis of the enrichments identified the taxonomy and metabolic potential of microbial community members. Long-read sequencing of the whole community DNA (Day 68) yielded over 2 and 0.5 million reads with N50 of 5.9 and 6.2 kb for R_4_ and R_32_, respectively, after quality filtering and trimming. Reads assembly resulted in 2747 and 2002 contigs with N50 of 151 and 240 kb. After binning, we recovered a total of 21 (R_4_) and 18 (R_32_) high-quality metagenome-assembled genomes (MAGs) with over 90% completeness and under 5% contamination (Supplementary Tables S7 and S8). The top 10 most abundant high-quality MAGs accounted for 78% (R_4_) and 57% (R_32_) of the mapped reads normalised to the corresponding MAG length (Fig. 3). We considered only the 10 most abundant high-quality MAGs for further analysis (Fig. 3), and grouped all low-abundant high-quality and all medium-quality MAGs (<90% completeness and >5% contamination) into ‘others’ (Supplementary Tables S7 and S8 and Supplementary Figs S11–S18). Low-quality bins (<70% completeness or >10% contamination) were grouped with the unbinned fraction, accounting for 18% (R_4_) and 26% (R_32_) of the community. MAG-based taxonomic analysis revealed two distinct communities, both dominated by the *Proteobacteria* phylum (Supplementary Tables S7 and S8). R_4_ was co-dominated by members of the *Denitromonas* (*Gammaproteobacteria*) and *Wagnerdoeblera* (*Alphaproteobacteria*) genera (Fig. 3). In R_32_, the two most abundant MAGs belonged to the *Castellaniella* genus (*Gammaproteobacteria*).

All high-quality MAGs contained at least one gene of the denitrification pathway, and full denitrifiers (with genes encoding for all denitrifications steps) dominated the community in R_32_ (Fig. 3 and Supplementary Section 5). The membrane-bound NO_3_^−^ reductase gene (*narGHI*) was annotated in most MAGs, whereas only a few also possessed the periplasmic reductase gene (*napAB*). Most MAGs had either a Cu-type (*nirK*) or *cd1*-type (*nirS*) NO_2_^−^ reductase gene, with some possessing both. Overall, the cytochrome c-dependent nitric oxide reductase genes (*norBC*) were more frequent than the quinol-dependent reductase genes (*norZ*). *norZ* in members of the *Castellaniella* genus were always accompanied with an additional *norB* gene. The N_2_O reductase gene (*nosZ*) was widespread in both reactors, and was dominated by the clade I type. No subunits of the ammonia monooxygenase (*amoABC*) and hydroxylamine oxidoreductase (*hao*) genes were found. Also, the *nrfAH* genes, catalysing the dissimilatory reduction of NO_2_^−^ to NH_4_^+^, were essentially absent in the MAGs (Supplementary Section 5). All denitrifying MAGs also contained the genes encoding the O_2_-reducing terminal oxidases (complex IV) (Supplementary Section 5), and enzymes protecting against reactive oxygen species (ROS), including superoxide dismutases (SODs) and catalases/peroxidases (Fig. 3).

### Highly comparable anoxic and oxic proteomic profiles

Shotgun metaproteomics of the steady-state enrichments (day 68) revealed the oxic and anoxic presence of key denitrification and ROS-protecting enzymes by each MAG (Fig. 3 and Supplementary Section 5). Over 70% (R_4_) and 50% (R_32_) of the detected total peptide intensity (peak area) uniquely matched with proteins predicted from the respective metagenomes. A total of 750/849 and 724/576 proteins of R_4_ and R_32_ (oxic/anoxic) were identified by at least two unique peptides. The protein-based relative abundance of most MAGs was consistent with their genome-based abundance (Fig. 3, right bar charts). The contribution to the overall proteome of the unbinned and others fraction combined, accounting for 22% and 43% of the metagenomes, was only 4% and 23% for R_4_ and R_32_, respectively.

The overall and MAG-specific relative abundances of the detected denitrification enzymes was highly comparable between the oxic and anoxic phase in each enrichment (Fig. 3 and Supplementary Section 5). The catalytic subunits of the membrane-bound NO_3_^−^ reductase (NarG), Cu-type (NirK) or *cd1*-type (NirS) NO_2_^−^ reductase, and N_2_O reductase (NosZ) were consistently present. NosZ I and NosZ II were both expressed in R_4_, but only NosZ I was detected in R_32_. In R_4_, the two most abundant MAGs (bin1.1 and bin1.2) accounted for most of the expressed denitrification proteins. On the contrary, in R_32_, lower abundant MAGs significantly contributed to the expression of NirS and NosZ. Moreover, NirS was the dominant type of NO_2_^−^ reductase detected in R_32_. The periplasmic NO_3_^−^ reductase (NapAB) was not detected in either of the communities (Fig. 3). With respect to oxygen, the abundance of the superoxide dismutase SOD2 and different catalases and peroxidases were detected primarily in the dominant MAGs (Fig. 3). The used protocol was not optimised for membrane-bound proteins, such as the cytochrome c- (cNor) and quinol-dependent (qNor) NO reductases, and the membrane-bound O_2_-reducing terminal oxidases (Cta, Cco, Cyo, Cyd) (Supplementary Section 5).

## Discussion

Two planktonic, nitrification-inhibited denitrifying communities co-respiring O_2_ and nitrogen oxides were enriched under alternating oxic/anoxic conditions at frequencies representative of both natural (e.g. coastal sediments [20]) and engineered (e.g. wastewater treatment, supplementary Section 6) ecosystems. Significant denitrification occurred at high oxygen concentrations, with almost 40% of the electrons from organic carbon being respired with NO_3_^−^ in the reactor with longer oxic/anoxic periods (R_4_). The high aerobic NO_3_^−^ reduction rates in this reactor—only half of the anaerobic rates—suggest the enrichment of a more O_2_-tolerant denitrifying community than under more frequent oxic/anoxic transitions (R_32_). Typically, the co-respiration of nitrogen oxides and oxygen is characterised in monocultures under continuous aeration, resulting in relatively low reported rates (as previously reviewed [10]). Only one study [21] emphasised the significance of alternating oxic/anoxic conditions for enhanced aerobic denitrification. However, most studies are based on a limited number of isolates, making their extrapolation to complex communities challenging. Few works quantified the contribution of aerobic heterotrophic denitrification in natural ecosystems with fluctuating oxic/anoxic conditions, namely, aggregate-forming extracted soil bacteria [25], sea sediments [24], and coastal sediments [20], yet at usually lower oxygen concentrations. The study with coastal sediments [20] reported peaks of aerobic NO_3_^−^ reduction rates up to 60% of the anaerobic rates at alternating oxic/anoxic conditions above 3-mg O_2_/L. However, only up to 5% of the electrons were respired via denitrification during aeration [20], and anoxic niches could not be completely ruled out in any of the abovementioned studies. Instead, microscopy confirmed that our cultures were planktonic (Supplementary Fig. S4) and the aeration overcapacity was 7.5-fold the actual respiration rates, so we can confidently exclude anoxic micro-niches to have significantly contributed to the overall rate. Besides, to maintain the high aerobic NO_3_^−^ conversion rate measured in R_4_, at least 40% of the active biomass would have had to be in anoxic micro-niches, which would have been unequivocally visible. Overall, we quantitatively show that aerobic denitrification is ecologically relevant in microbial communities exposed to O_2_ fluctuations. Furthermore, we estimated that on average 12% (R_4_) and 24% (R_32_) of NO_3_^−^ was emitted as N_2_O during aeration, highlighting that heterotrophic denitrification also holds the potential to be a major contributor to aerobic N_2_O emissions.

The oxic and anoxic proteomic profiles were nearly identical within each enrichment. The three most abundant MAGs in R_4_ and R_32_ accounted for 90% and 68% of the respective proteomes, proving their prominent functional role. All denitrification enzymes remained present and, at least partially, active under oxic conditions. In contrast, in continuous monocultures, most denitrifying proteins are generally detected exclusively in anaerobically grown cells, and their abundance and activity is negligible under solely oxic conditions [7, 70, 71]. Traditionally, oxygen is believed to suppress the transcription of denitrifying genes [7, 9, 72], even if denitrification transcripts have also been detected during aeration (for example, *narG* and *nosZ* at 100 μM O_2_ [8]; *narG, norB*, and *nosZ* at 235 μM O_2_ [72, 73]). Besides, prolonged exposure to alternating conditions has been hypothesised to reduce the direct impact of O_2_ [18, 20, 21, 25]. We worked at oxic/anoxic transition frequencies significantly higher than the imposed growth rates, i.e. the O_2_ cycling was faster than protein turnover. Consequently, denitrifying enzymes synthesised in the anoxic period most likely persisted and remained active in the oxic phase, masking the influence of any potential oxygen-mediated transcriptional regulation on protein abundances. Yet, it would be of interest to determine the protein regulation mechanisms of denitrifying organisms under highly dynamic oxygen conditions. From an ecological perspective, open culture cultivation as applied here selects, by design, for the organisms that are the fittest for the imposed conditions [74]. Therefore, we postulate that organisms capable of maintaining a significant denitrification activity in the presence of oxygen can outcompete (i.e. have a competitive advantage over) other heterotrophs in environments with fluctuating oxygen availabilities. In analogy, relevant aerobic residual denitrification potentials are to be expected in environments with rapid O_2_ fluctuations, such as sediments [20] and wastewater treatment plants (Supplementary Section 6).

The lower aerobic denitrification rates, compared to the anaerobic ones, can thus reasonably be ascribed to reversible enzyme inhibition or electron competition with O_2_, rather than to transcriptional or translational regulation [8, 10, 75]. The O_2_ impact differed for each denitrification step, in line with previous observations [7, 76]. Even though NO_2_^−^ and NO were hardly detected, N_2_O consistently accumulated, possibly as a result of the often reported higher relative oxygen sensitivity of NosZ [25, 76, 77]. The marked N_2_O accumulation at the onset of anoxia implies a slower post-aerobiosis recovery of Nos compared to the other reductases. The progressive N_2_O accumulation under full aeration suggests a gradual yet incomplete inhibition of N_2_O reduction, as previously observed [8]. In fact, we estimated that 80%–90% of the produced N_2_O was still reduced during aeration. Based on such a high N_2_O consumption, one may argue that heterotrophic denitrification could function as a sink for nitrifier-produced N_2_O during intermittent oxic conditions. However, N_2_O did accumulate, indicating higher production than consumption rates, and suggesting that aerobic denitrification likely acts as a net N_2_O source rather than a sink in dynamic O_2_ environments. Unexpectedly, N_2_O accumulation fluctuated throughout the operational steady-state of both reactors despite the consistency of all other conversion rates. N_2_O accumulation results from the unbalance between its production and consumption rates. Minor variations in the latter two lead to significant fluctuations in the comparably lower net N_2_O accumulation. Such fluctuations may result from stochastic micro-oscillations in microbial composition, as documented in functionally redundant communities [78–80]. Taken together, these results highlight the need for more research on the impact of variable O_2_ availability on denitrification and, from a physiological perspective, further support the long-term competitive advantage of metabolic preparedness in dynamic environments.

Contrary to the long-standing assumption that the periplasmic reductase Nap is required for aerobic nitrate respiration [20, 21, 23, 25, 81], only the membrane-bound Nar was detected in our metaproteomes. Although preferential extraction or sequencing, and biases towards more abundant species can impact protein recovery [82], both Nap subunits are soluble [83] and are usually detected with equivalent protocols (e.g. in *Paracoccus denitrificans* [71]). Also, the *napAB* genes were found in the most abundant MAGs, e.g. bin1.1 accounting for 50% of the proteome in R_4_. Therefore, although the presence of Nap at very low abundance cannot be completely ruled out, NO_3_^−^ reduction in our cultures was evidently driven by Nar and thus contributed directly to proton translocation under oxic conditions. Studies on pure cultures of *P. pantotrophus* and *P. denitrificans* reported Nar and Nap to be preferentially expressed under continuous anoxic or oxic conditions, respectively [71, 81, 84]. The excess NO_3_^−^ in our cultures may have alleviated the potential oxygen inhibition of NO_3_^−^ uptake [85, 86], favouring the lower-affinity Nar over Nap [87]. However, high levels of *nap* transcription and Nap activity were measured in *P. pantotrophus* grown in oxic NO_3_^−^ excess chemostats [88], suggesting that factors other than NO_3_^−^ affinity determined the preferential Nar expression in our enrichments. Overall, the here observed consistent and exclusive expression of Nar suggests a higher versatility under alternating oxic/anoxic conditions, and challenges the use of *nap* as specific marker gene for aerobic heterotrophic denitrification [19, 20].

The subsequent nitrogen oxides reduction steps featured different degrees of labour division among the MAGs in the two enrichments. Both nitrite reductases (NirK and NirS), and both clade I and II N_2_O reductases (NosZ) were primarily expressed by the dominant MAGs in R_4_. Conversely, the proteomic profile of R_32_ revealed a more prominent role of lower abundant MAGs in NO_2_^−^ and N_2_O reduction. Also, despite the widespread presence of the *nirK* gene in R_32_, mainly NirS was expressed. The preferential expression of NirK in R_4_ and NirS in R_32_ may account for the conflicting accumulation of nitrite in the anoxic (R_4_) and oxic (R_32_) phases (Supplementary Fig. S8). Although O_2_-driven preferential expression and activity of either NirK or NirS is plausible, conflicting O_2_-sensitivities have been reported [76], warranting further research on the determinants of functional homologues preferences. The expression of NirK and NirS by several MAGs without nitrate reductase may explain the low nitrite accumulation in both cultures. In line with previous proteomic studies [71, 89], the detection of the membrane-bound hydrophobic qNor and cNor, intrinsically challenging to detect in proteomic analyses, was negligible. The *nosZ I* was annotated in most MAGs, with many expressing the encoded NosZ I. In turn, NosZ II was exclusively detected in R_4_. It is here tempting to speculate that the higher aerobic denitrification rates in R_4_ related to the reported lower O_2_ inhibition of clade II NosZ [73]. However, these observations were limited to one *nosZ II*-harbouring *Azospira* strain and no evident clade-dependent differences in O_2_-tolerance were observed in a more recent study [90]. Furthermore, different physiological mechanisms such as strain-specific ability to scavenge O_2_ may impact the O_2_-tolerance of N_2_O-reducers [90].

In conclusion, beyond decades of research based on pure cultures, we show that organisms capable of co-respiring nitrogen oxides and oxygen have a competitive advantage in complex ecosystems exposed to time-varying oxygen availabilities. We posit that the aerobic denitrification rates, comparable to the anaerobic ones, likely resulted from the activity of anaerobically produced enzymes, as the imposed oxic/anoxic frequencies exceeded the organisms growth rate, a scenario often observed in natural and engineered microbiomes. Our findings also suggest that heterotrophic denitrification may be an important aerobic N_2_O source alongside nitrification in O_2_-fluctuating environments.

## Supplementary Material

20240702_AerDEN_SI_wrae116

## Data Availability

Raw DNA reads were deposited on the NCBI Sequence Read Archive and medium- and high-quality MAGs were deposited in Genbank under BioProject PRJNA977937. Mass spectrometric raw data and unprocessed search files are publicly available via the PRIDE repository under the project code PXD042057.
