# Supplementary material for: Aerobic denitrification as an N2O source from microbial communities
Source: ISME J. 2024 Jun 24;18(1):wrae116. doi: 10.1093/ismejo/wrae116 (PMC11272060; doi:10.1093/ismejo/wrae116)
Supplement: 20240702_AerDEN_SI_wrae116 [file 20240702_aerden_si_wrae116.pdf]

Supplementary information

Aerobic denitrification as an N_2O source from microbial communities

Nina Roothans[a], Minke Gabriëls[a], Thomas Abeel[b,c], Martin Pabst[a], Mark C.M. van Loosdrecht[a], Michele Laureni[d,*]

[a] Department of Biotechnology, Delft University of Technology, van der Maasweg 9, 2629 HZ Delft, the Netherlands
[b] Delft Bioinformatics Lab, Delft University of Technology, van Mourik Broekmanweg 6, Delft 2628 XE, the Netherlands
[c] Infectious Disease and Microbiome Program, Broad Institute of MIT and Harvard, 415 Main Street, Cambridge, MA 02142, United States of America
[d] Department of Water Management, Delft University of Technology, Stevinweg 1, 2628 CN Delft, the Netherlands

*Corresponding author: Michele Laureni, Department of Water Management, Delft University of Technology, Stevinweg 1, 2628 CN Delft, the Netherlands. Email: m.laureni@tudelft.nl

Contents

1. Reactor operation....2
2. Calculation of consumption and production rates 5
3. Nitrification assays 15
4. Metagenomics....16
5. Heatmaps with gene presence and protein expression 20
6. Oxic/anoxic cycling in 5 reference Dutch WWTPs 24
7. Microbial composition of the inoculum 26

1. Reactor operation

Table S1. Measured average substrate loading and steady-state conversion rates of the low- (R_4) and high-frequency (R_{32}) reactors. Overall rates refer to rates estimated over the total duration of an oxic/anoxic cycle, and considers the average of three effluent concentrations (beginning and end of oxic phase, and end of anoxic one). Only for the gaseous compounds (CO_2 and N_2O) individual rates for each phase (oxic and anoxic) were measured on top of the overall rates. O_2 was only added and consumed in the oxic phase, yet an "overall" rate was also calculated by averaging the aerobic O_2 consumption over the entire cycle duration (eq. S9) for further balancing purposes.

			Loading		Conversion	
Compound	Phase	Units	R_4	R_{32}	R_4	R_{32}
CO_2	Oxic	C-mmol/h	-	-	1.37 ± 0.07	1.5 ± 0.1
	Anoxic	C-mmol/h	-	-	1.15 ± 0.08	1.4 ± 0.1
	Overall	C-mmol/h	-	-	1.30 ± 0.06	1.5 ± 0.1
N_2O	Oxic	N-mmol/h	-	-	0.06 ± 0.04	0.04 ± 0.03
	Anoxic	N-mmol/h	-	-	0.04 ± 0.04	0.04 ± 0.02
	Overall	N-mmol/h	-	-	0.05 ± 0.03	0.04 ± 0.03
NO_3^-	Overall	N-mmol/h	0.96 ± 0.02	0.91 ± 0.02	-0.73 ± 0.08	-0.60 ± 0.04
NO_2^-	Overall	N-mmol/h	-	-	0.03 ± 0.05	0.01 ± 0.02
NH_4^+	Overall	N-mmol/h	0.49 ± 0.02	0.47 ± 0.01	-0.22 ± 0.02	-0.27 ± 0.04
O_2	Oxic	mmol/h	210	210	-1.0 ± 0.1	-1.6 ± 0.2
	"Overall"	mmol/h	141	149	-0.70 ± 0.07	-1.2 ± 0.2
Biomass[a]	Overall	C-mmol/h	-	-	1.08 ± 0.09	1.4 ± 0.2
Acetate[b]	Overall	C-mmol/h	0.85 ± 0.02	1.02 ± 0.01	-0.85 ± 0.02	-1.02 ± 0.01
Propionate[b]	Overall	C-mmol/h	0.91 ± 0.02	1.09 ± 0.01	-0.91 ± 0.02	-1.09 ± 0.01
Butyrate[b]	Overall	C-mmol/h	0.68 ± 0.02	0.82 ± 0.01	-0.69 ± 0.02	-0.83 ± 0.01

[a] Calculated from the NH_4^+ consumption rates.

[b] Always 0 in the effluent.

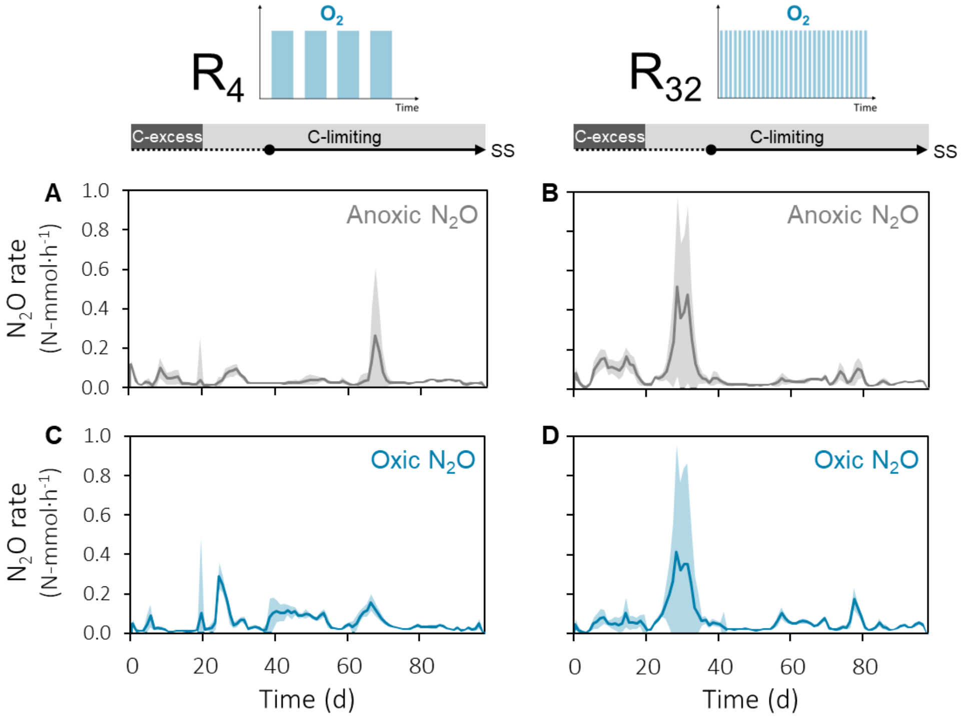

Figure S1. Daily average anoxic (top, grey) and oxic (bottom, blue) N_2O production rates in the low- (R_4) and high-frequency (R_{32}) reactors. The shaded areas are the standard deviation of the daily averages, representing the fluctuation of N_2O rates within each day.

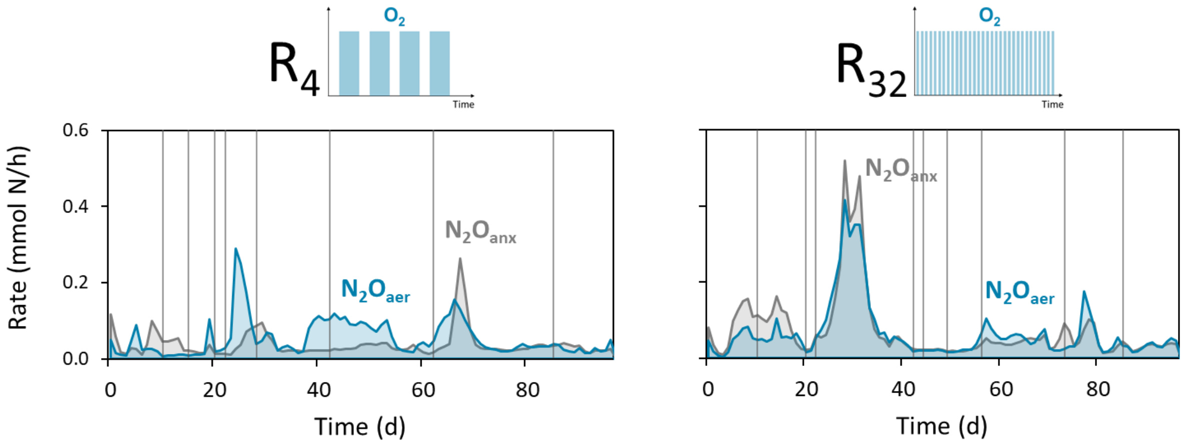

Figure S2. Headspace wall-growth cleaning events (vertical lines) did not affect the profile of the oxic (blue) and anoxic (grey) N_2O emissions in the low- (R_4) and high-frequency (R_{32}) reactors.

Table S2. Nitrite concentrations throughout the steady-state period.

R_4		R_{32}	
Day	NO_2^- (mM)	Day	NO_2^- (mM)
37	0.3 ± 0.3	37	0.0004 ± 0.0009
41	0.6 ± 0.4	41	0.02 ± 0.02
43	0.8 ± 0.3	44	0.04 ± 0.01
49	8.0 ± 0.2	49	0.05 ± 0.02
55	0.2 ± 0.2	55	0.83 ± 0.01
68	0.2 ± 0.3	68	2.85 ± 0.06

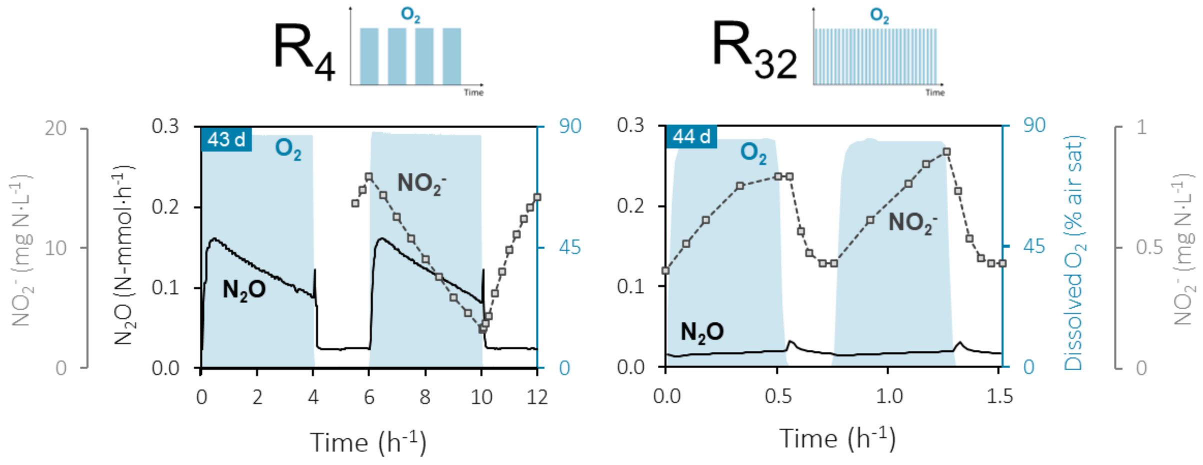

Figure S3. Concentration profiles during one or two oxic/anoxic cycles after 43 (R_4) or 44 (R_{32}) days of operation. The nitrite (symbols) and dissolved oxygen concentrations (blue area), as well as the N_2O production rate measured every minute (black line) are represented. In this case, nitrite accumulated in the anoxic phase in R_4 and in the oxic phase in R_{32}.

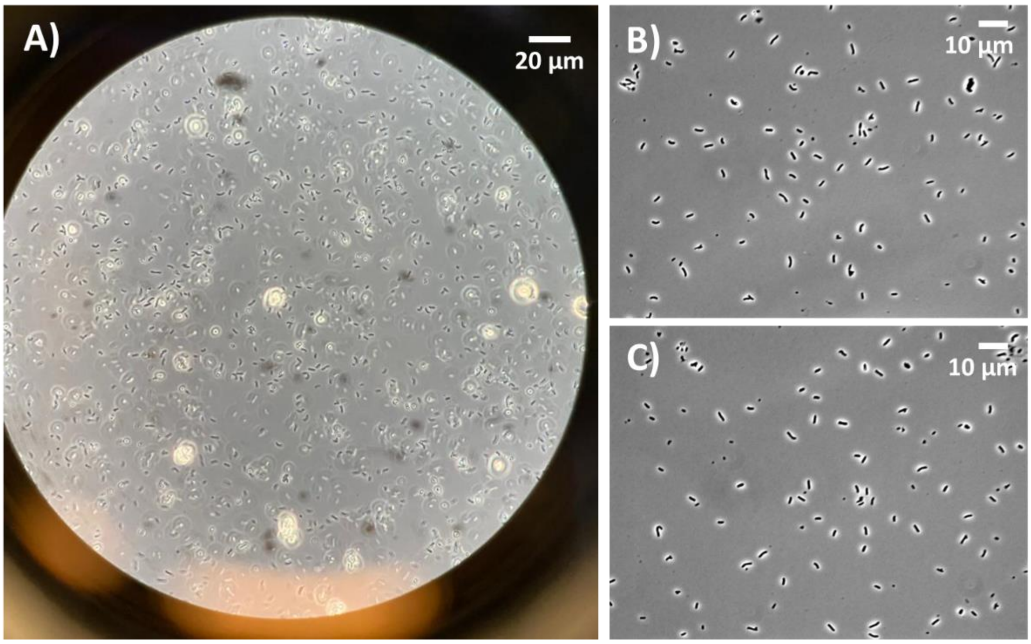

Figure S4. Microscopic pictures of the broth of R_4. (A) Undiluted culture after 35 days of operation, 400x amplified. (B, C) 8-fold diluted culture after 55 days of operation, 1000x amplified.

k_La determination. The oxygen volumetric mass transfer coefficient (k_La) was determined to calculate the oxygen transfer rate in the oxic phase. The k_La of R_4 and R_{32} were determined under identical conditions as the enrichments (500 rpm stirring, 400 mL/min gas flow), but with water instead of biomass. The O_2 transfer rates were determined by following the dissolved oxygen concentration during the sparging of air (400 mL/min) in anoxic water. The k_La was obtained by fitting the integrated mass transfer equation to the dissolved O_2 concentration profile over time, with C_{O2}^* the solubility of O_2 at 20°C:

$$C_{O_2} = C_{O_2}^* \cdot (1 - e^{-k_L a \cdot t}) \qquad \text{(eq. S1)}$$

The obtained k_La values were 36.2 (R_4) and 37.0 h^{-1} (R_{32}).

2. Calculation of consumption and production rates

Consumption and production rates of all dissolved and gaseous compounds were measured or estimated in the oxic and anoxic phases, and overall (combined oxic and anoxic). Consumption rates are negative and production rates are positive.

Overall consumption and production rates in the liquid. Consumption and production rates of NO_3^-, NO_2^- ($C_{in} = 0$), NH_4^+ and the organic compounds acetate, propionate and butyrate ($C_{out} = 0$) were calculated from a mass balance:

$$R_i(\mathrm{mmol \cdot h^{-1}}) = F_{i,out} \cdot C_{i,out} - F_{i,in} \cdot C_{i,in} \qquad \text{(eq. S2)}$$

with R_i the molar rate (mmol·h^{-1}), F_i the influent and effluent flow rates (L·h^{-1}), and C_i the concentration of compound i (mmol·L^{-1}). C_{out} was the average of three effluent measurements (taken at the beginning and end of the oxic phase, and end of the anoxic phase). The sample for C_{in} was taken directly at the entry point of the reactors, yet differences with stock feed solution remained negligible throughout the experimental period. The flow rates were the average of the measured flow rates during the entire operation. Linear error propagation was applied to determine the standard deviation in the rates (eq. S3), using the standard deviations of F_{in}, F_{out}, and C_{out} (deviation between the three measurements).

Linear error propagation of a function f dependent on multiple variables (x, y, …):

$$f(x, y, \ldots): \sigma_f = \sqrt{\left(\frac{\partial f}{\partial x}\right)^2 \cdot \sigma_x^2 + \left(\frac{\partial f}{\partial y}\right)^2 \cdot \sigma_y^2 + \cdots} \qquad \text{(eq. S3)}$$

with σ_f, σ_x, and σ_y the standard deviations of f, x, and y, respectively, and $\partial f/\partial x$ and $\partial f/\partial y$ the partial derivatives of f with respect to x and y, respectively.

Overall, aerobic, and anaerobic consumption and production rates in the gas phase. A script was written in RStudio to calculate the overall, and separate aerobic and anaerobic N_2O and CO_2 rates from continuous measurements recorded every minute. The molar gas flow leaving the reactor was calculated for each time point based on the constant influent volumetric gas flow rate (400 mL·min^{-1}) and the measured temperature and atmospheric pressure:

$$N_{gas}(\mathrm{mmol \cdot h^{-1}}) = \frac{P_{atm} \cdot F_{V,gas}}{R \cdot T} \qquad \text{(eq. S4)}$$

With N_{gas} the molar gas flow rate (mmol·h^{-1}), P_{atm} the atmospheric pressure (mbar), $F_{V,gas}$ the volumetric gas flow rate, R the ideal gas constant (L·mbar·K^{-1}·mmol^{-1}), and T the reactor temperature (K). The molar gas fractions were normalized to the zero measurement before further calculations, by subtracting the corresponding value measured for the zero concentration. For each of the gases, the molar flow rates in the off-gas were calculated at each time point from measured gas fractions and the total molar gas flow rate:

$$N_{CO_2}(\mathrm{mmol \cdot h^{-1}}) = y_{CO_2} \cdot N_{gas} \qquad \text{(eq. S5)}$$

The N_2O rate was normalized per mole of nitrogen:

$$N_{N_2O}(\mathrm{Nmmol \cdot h^{-1}}) = 2 \cdot y_{N_2O} \cdot N_{gas} \quad \text{(eq. S6)}$$

with N_i the molar gas flow rates (mmol·h^{-1}), N_{gas} the molar gas flow rate (mmol·h^{-1}), and y_i the molar fractions of each compound in the off-gas. The accumulation rates at every time point were calculated with the following mass balance:

$$R_{CO_2,N_2O}\ ((\mathrm{N})\mathrm{mmol \cdot h^{-1}}) = N_{i,out} - N_{i,in} \quad \text{(eq. S7)}$$

The average fraction of CO_2 in the influent air was 450 ppm. The dataset containing the rates at every minute was split in oxic and anoxic periods, with the oxic period defined for time points with DO > 1% (0.08 mg O_2·L^{-1}). Daily average aerobic, anaerobic, and overall rates were calculated with the corresponding dataset. The standard deviation of these averages was taken as the uncertainty in the rates.

Overall and aerobic consumption and production rates of oxygen. The O_2 consumption rates during the oxic phase were calculated from the dissolved oxygen measurements during maximum aeration periods (> 20% O_2 in the off-gas and dissolved oxygen > 70%):

$$R_{O2}(\mathrm{mmol \cdot h^{-1}}) = k_La \cdot H_{O_2} \cdot P_{atm} \cdot y_{O_2} \cdot (1 - DO) \cdot V \quad \text{(eq. S8)}$$

With k_La the experimentally measured transfer coefficient (h^{-1}), H_{O2} the Henry coefficient for O_2 (0.001283 mmol·L^{-1}·mbar^{-1}), P_{atm} the atmospheric pressure (mbar), y_{O2} the O_2 molar fraction in the off-gas, DO the measured dissolved oxygen, and V the broth volume (L).

The aeration over-capacity in the reactors was determined by comparing the maximum O_2 transfer rate from the gas to the liquid (equivalent to the maximum possible O_2 microbial respiration rate) to the actual O_2 respiration rates. The maximum possible O_2 transfer rates, *i.e.* the maximum microbial respiration capacity, would be achieved when the DO is 0, so eq. S8 can be simplified into eq. S9. These rates were calculated to be 7.5-fold higher than the actual O_2 respiration rates, reflecting the aeration over-capacity in the reactors.

$$R_{O2}^{max}(\mathrm{mmol \cdot h^{-1}}) = k_La \cdot H_{O_2} \cdot P_{atm} \cdot y_{O_2} \cdot V \quad \text{(eq. S9)}$$

Daily averages were calculated and taken for further calculations. The standard deviation of these averages were taken as the uncertainty of the rates. The “overall” consumption rate of O_2 for further electron balancing purposes over an entire cycle was taken as the weighted average of the aerobic and anaerobic (=0) rates:

$$R_i^{overall} = \frac{t_{aerobic}}{24} \cdot R_i^{aerobic} + \frac{t_{anaerobic}}{24} \cdot R_i^{anaerobic} \quad \text{(eq. S10)}$$

with $t_{aerobic}$ and $t_{anaerobic}$ (h) the total time in one day in which the dissolved oxygen was above or below 1%, respectively.

Overall respiratory electron flow to nitrogen oxides and O_2. The absolute and relative overall flows of electrons from organic electron donors to the electron acceptors NO_3^- and O_2 were calculated from the overall rates considering four and five electrons for the conversion of NO_3^- to N_2O and N_2, respectively, and four electrons for the reduction of O_2 to H_2O (Table S3). NO_3^- and O_2 were the sole electron acceptors and NH_4^+ fully sustained biomass growth (detailed in the following section), minimizing NO_3^- assimilation. Thus, both substrates account for the entirety of the catabolic electron flow.

Table S3. Absolute (mmol e^-/h) and relative (%) overall electron flows from organic carbon to the electron acceptors NO_3^- and O_2 in the low- (R_4) and high-frequency (R_{32}) reactors. The electron flows were calculated from the NO_3^- and O_2 consumption and the N_2O accumulation rates.

Electron flow	e^-	R_4 (mmol e^-/h)	R_{32} (mmol e^-/h)
$NO_3^- \rightarrow N_2O$	4	0.2 ± 0.1	0.2 ± 0.1
$NO_3^- \rightarrow N_2$	5	3.4 ± 0.4	2.8 ± 0.2
$O_2 \rightarrow H_2O$	4	2.8 ± 0.3	4.7 ± 0.6
% NO_3^- / Total		56 ± 4%	39 ± 4%
% O_2 / Total		44 ± 4%	61 ± 4%

Biomass production rates. The biomass concentration was estimated from NH_4^+ measurements and carbon balances. In the studied system, ammonia oxidation was fully inhibited via continuous ATU addition, thus the assimilation into biomass (0.2 N-mol/C-mol) was the sole NH_4^+ consumption process. The biomass production rate was calculated as follows:

$$R_X = \left|R_{NH_4^+}/0.2\right| \qquad \text{(eq. S11)}$$

The carbon balance included only the organic carbon substrates (acetate, propionate, and butyrate), CO_2 and biomass, as no other products were detected in the HPLC. Therefore, the biomass production rate could also be directly calculated according to the following equation:

$$R_X\ (\text{Cmmol} \cdot h^{-1}) = \left|R_{Ace} + R_{Pro} + R_{But} + R_{CO2}\right| \qquad \text{(eq. S12)}$$

For all calculations, an empirical biomass formula of $CH_{1.8}N_{0.2}O_{0.6}$ was used. The biomass concentration (C_X in C-mmol·L^{-1}) was then estimated from the production rate (R_X) and the flow rate (F_{out} in L·h^{-1}):

$$C_X = R_X/F_{out} \qquad \text{(eq. S13)}$$

Both methods resulted in similar estimations, showing that the biomass concentration and its production rate can be determined through either one of the methods (Figure S5). The biomass rates and concentrations based on NH_4^+ measurements were used for further calculations. Error propagation was applied to determine the standard deviation in the rates, using the standard deviations of F_{out}, and NH_4^+, organic carbon, and CO_2 rates.

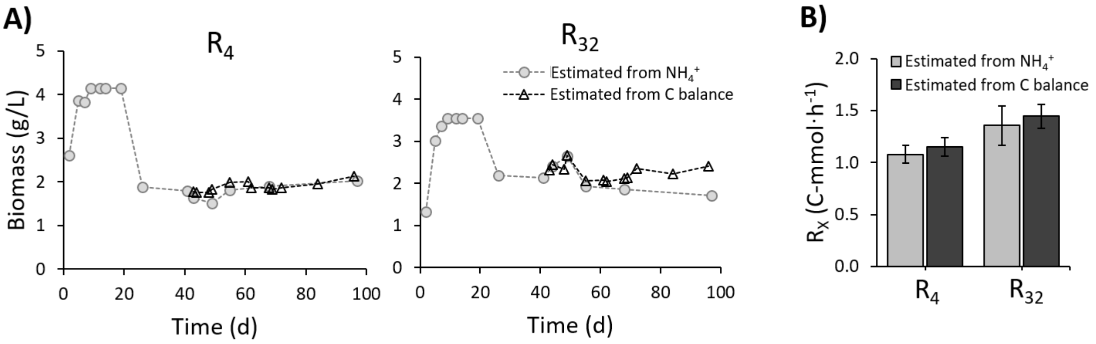

Figure S5. Highly comparable biomass concentrations and production rates in the low- (R_4) and high-frequency (R_{32}) reactors estimated with two different methods. Panel A: Biomass concentration over time in both the low- and high-frequency reactors, expressed as g/L. **Panel B:** average biomass production rates during the steady-state. The estimated concentrations and rates were determined from the NH_4^+ measurements (light grey) and the carbon balance (dark grey), i.e. the organic substrate and CO_2 measurements.

Overall carbon, nitrogen, and electron balances. Mass balances were performed using the consumption and production rates averaged over the steady-state period to ensure that all substrates and products were recovered. The overall carbon balance was calculated from the consumption and production rates of acetate, propionate, butyrate, biomass (estimated from the NH_4^+ rates), and CO_2:

$$\text{C balance (\%)} = \frac{R_{in}}{R_{out}} = \frac{|R_{Ace}+R_{Pro}+R_{But}|}{R_X+R_{CO_2}} \qquad \text{(eq. S14)}$$

The nitrogen compounds involved in the nitrogen balance would be NH_4^+, biomass, NO_3^-, NO_2^-, NO, N_2O, and N_2. All compounds were measured (or estimated, in the case of biomass) except N_2. NO and NO_2^- accumulation was absent or negligible throughout the entire experiment, so we could assume that the missing nitrogen was recovered as N_2, representing full denitrification from NO_3^-:

$$R_{N_2} = |R_{NO_3^-}| - R_{N_2O} \qquad \text{(eq. S15)}$$

Based on all calculated and estimated rates, an electron balance was calculated.

$$e^- \text{ balance (\%)} = \frac{R_{eD}}{R_{eA}} = \frac{|4 \cdot R_{Ace}+4.7 \cdot R_{Pro}+5 \cdot R_{But}|}{-8 \cdot R_{NO_3^-}-4 \cdot R_{N_2O}-3 \cdot R_{N_2}-4 \cdot R_{O_2}+4.2 \cdot R_X} \qquad \text{(eq. S16)}$$

Uncertainty of the balances were calculated through linear error propagation from the standard deviations of the respective rates (eq. S3).

Table S4. Overall carbon, and electron balances over the entire steady-state period of the low- (R_4) and high-frequency (R_{32}) oxic/anoxic cycling denitrifying reactors.

**Reactor**	**Low-frequency**	**High-frequency**
Carbon balance	103 ± 5%	101 ± 9%
Electrons balance	103 ± 8%	100 ± 8%

Estimation of the NO_3^- consumption and production rates in the oxic and anoxic phases. Separate aerobic and anaerobic rates were calculated for all compounds continuously measured in the gas (N_2O and CO_2) or liquid phase (O_2). In turn, grab samples for the quantification of all other compounds were less sensitive to the small concentration changes occurring during each phase, so consumption and production rates could not be determined directly with high confidence. Instead, aerobic and anaerobic rates were calculated from the overall mass balance and the phase-specific N_2O, CO_2, and O_2 rates as detailed below. In short, as the overall balances closed (Table S4), all biological processes taking place in the controlled environments of the reactor are known. Also, the overall rates are the sum of the aerobic and anaerobic ones weighted by their corresponding time fractions.

The calculation of aerobic and anaerobic conversion rates detailed below were based on carbon, nitrogen, and electron balances, so one needs to know which processes occurred in the reactor broth in each phase. Specifically, from the closed carbon and electron balances (Table S4) we know that denitrification occurred, with N_2O and N_2 as end-products. We do not know, however, which fraction of this conversion occurred in the oxic and anoxic phases. To determine this, three scenarios were considered (Figure S6). The rationale underlying these scenarios is briefly explained:

1) **Scenario 1** was developed based on past literature. Aerobic denitrification was widely considered to be absent or negligible under fully oxic conditions. Nevertheless, we measured aerobic production of N_2O in our reactors, which means that at least part of the NO_3^- was aerobically converted to N_2O. For this scenario, we assumed that this was the only fraction of NO_3^- converted aerobically, with the remaining converted under anoxic conditions.
2) The aerobic and anaerobic electron balances did not close in **scenario 1**, which means that our assumption was incorrect.
3) Based on the electron gaps observed in scenario 1, we developed **scenario 2**. In this case, we rationalized that the missing or surplus of electrons in scenario 1 must belong to a "blind" amount of NO_3^- aerobically reduced to N_2. In other words, for **scenario 2**, we considered that part of the NO_3^- was aerobically converted to N_2O (directly measured, same as scenario 1) and an additional part was converted to N_2 (estimated).
4) The estimations made in **scenario 2** were validated with measurements, so we could confidently estimate the aerobic and anaerobic NO_3^- consumption rates.
5) Even though scenario 2 seems to accurately describe the microbial conversions in our reactors, we considered the possibility of PHA accumulation in the anoxic phase. **Scenario 3** was developed to evaluate if the potential PHA accumulation would affect the estimated aerobic and anaerobic NO_3^- consumption rates in scenario 2. We concluded that even large amounts of PHA accumulation would not affect the estimated rates.

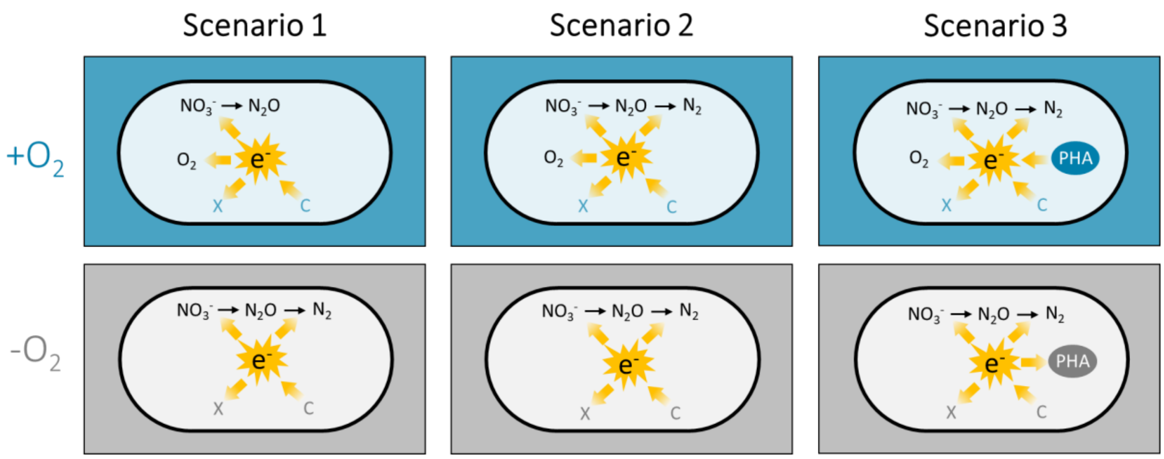

Figure S6. Schematic representation of the three scenarios considered to calculate the aerobic and anaerobic conversion rates of soluble substrates. The compounds/conversions included in the electron balance in each scenario in the anoxic (grey) and oxic (blue) conditions are represented. All scenarios considered the organic carbon © as electron donor and biomass (X) production as electron sink. Under oxic conditions, all scenarios considered O_2 reduction to H_2O. Under anoxic conditions, all scenarios considered full denitrification from NO_3^- to N_2. The different conversions considered for each scenario under oxic conditions were: (1) partial denitrification of NO_3^- to N_2O, no PHA pool; (2) full denitrification of NO_3^- to N_2, no PHA pool; (3) full denitrification of NO_3^- to N_2 with consumption of a PHA pool generated under anoxic conditions. The different conversions considered under anoxic conditions were: (1) and (2) no PHA pool; (3) PHA accumulation. We had experimental measurements of the N_2O and CO_2 production and O_2 consumption rates in each phase, in addition to the overall consumption and production rates of all compounds.

Detailed calculations performed in each scenario are also explained:

- **Scenario 1: <u>no</u> aerobic conversion NO_3^- to N_2, only to N_2O.** From literature, it is known that aerobic denitrification is not commonly observed in a denitrifying microbial community, at least not at a significant rate. However, in this study, significant N_2O production was observed during the aerated periods. So, at first, the aerobic NO_3^- consumption rate was assumed equal to the observed N_2O production, excluding any N_2 production:

$$R_{NO_3^-}^{aerobic} = -R_{N_2O}^{aerobic} \qquad \text{(eq. S17)}$$

The anaerobic NO_3^- consumption rate was calculated from the measured overall rate and the supposed aerobic rate, knowing that the overall consumption rate comes from a balance between the aerobic and the anaerobic rates (eq. S10). The N_2 production rate in the anoxic phase was calculated from the NO_3^- and the N_2O rates (eq. S15). Similarly to NO_3^-, the NH_4^+ consumption rates could not be determined in each phase individually. Therefore, differently from the overall mass balance approach, the biomass production rates in each phase were derived from the carbon mass balances (eq. S12). The validity of this estimation was proven above (Figure S5). The NH_4^+ consumption rate was then estimated from the biomass production rate (eq. S11).

Table S5. Summarized explanation of how the overall, aerobic, and anaerobic rates were determined in scenario 1.

Compound	Overall rate	Aerobic rate	Anaerobic rate
Organic substrate	Measured	Measured	Measured
CO_2	Measured	Measured	Measured
N_2O	Measured	Measured	Measured
O_2	Measured	Measured	Measured (= 0)
NH_4^+	Measured	Estimated from aerobic biomass	Estimated from anaerobic biomass
Biomass	Estimated from overall NH_4^+ (= C balance)	Estimated from aerobic C balance	Estimated from anaerobic C balance
NO_3^-	Measured	Estimated from aerobic N balance (= N_2O)	Estimated from aerobic and overall NO_3^- (eq. S10)
N_2	Estimated from overall N balance	Assumed to be 0	Estimated from anaerobic N balance

The electron balance (eq. S16) and electron gap were then calculated for both the oxic and anoxic phases (Table S6):

$$e^- \text{ gap} = R_{eD} - R_{eA} \qquad \text{(eq. S18)}$$

Table S6. Electron balances and gaps in the oxic and anoxic phases of the low-frequency and high-frequency reactors, assuming the exclusive conversion of NO_3^- to N_2O under oxic conditions.

**Reactor**	**Low-frequency**		**High-frequency**	
Phase	Oxic	Anoxic	Oxic	Anoxic
Electrons balance	124 ± 9%	69 ± 10%	105 ± 7%	81 ± 7%
Electron gap (e-mmol·h^{-1})	2.1 ± 0.7	-5.0 ± 2.3	0.7 ± 0.8	-3.2 ± 1.4

The electron balances did not close in either of the phases in both reactors. The balances show an underestimation of electrons accepted in the oxic phase and an overestimation in the anoxic phase. This suggests that more NO_3^- was reduced in the oxic phase than accounted for, whereas an excess NO_3^- reduction was accounted for in the anoxic phase.

Scenario 2: <u>yes</u> aerobic conversion of NO_3^- to both N_2 and N_2O - closing the electron mass balance. From the electron balances in the previous scenario and the symmetric electron gaps of the two phases, it was hypothesized that the surplus of reduced NO_3^- accounted for in the anoxic phase was actually reduced in the oxic phase. New aerobic and anaerobic NO_3^- consumption rates were estimated by closing the respective electron gaps, assuming full conversion of NO_3^- to N_2 (5 e^- transfer):

$$R_{NO_3^-}^{\text{Scenario 2}} = R_{NO_3^-}^{\text{Scenario 1}} - \frac{e^-_{gap}}{5} \qquad \text{(eq. S19)}$$

Linear error propagation was applied to estimate the standard deviations of the derived rates (eq. S3). With the estimations of the aerobic and anaerobic NO_3^- conversion rates,

we estimated the overall NO_3^- consumption rate (eq. S10). Considering that we also have the actual measured value for this rate, we could validate the calculation of the aerobic and anaerobic NO_3^- consumption rates by comparing the recalculated overall NO_3^- consumption rates in scenario 2 (eq. S10) to the measured rates (Figure S7, panel B). Therefore, we confidently estimated the aerobic and anaerobic NO_3^- consumption rates as 0.48 ± 0.14 and 1.13 ± 0.54 N-mmol/h (R_4) and 0.17 ± 0.17 and 1.34 ± 0.31 N-mmol/h (R_{32}), respectively. From the total aerobic electron flow, 36±7% and 11±11% went to denitrification.

These values were validated with direct calculations from measured concentration profiles throughout each phase, including the NO_2^- accumulation rates (supplementary Figures S8-9).

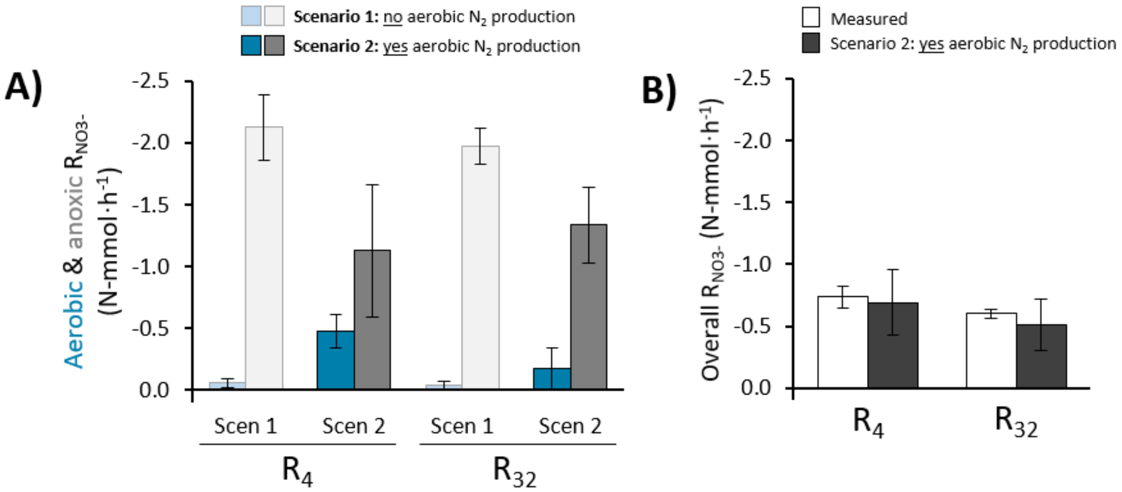

Figure S7. Nitrate consumption rates in the oxic (blue) and anoxic (grey) phases of the low- (R_4) and high-frequency (R_{32}) reactors. Panel A: comparison between the predicted rates according to scenario 1 (light, assuming <u>no</u> aerobic N_2 production) and scenario 2 (dark, assuming <u>yes</u> aerobic N_2 production). **Panel B:** The measured and estimated overall nitrate consumption rates were also compared to validate the calculations.

- **Scenario 3: <u>yes</u> aerobic conversion of NO_3^- to both N_2 and N_2O, and simultaneous PHA accumulation.** Cyclic conditions may select for populations accumulating storage compounds, such as polyhydroxyalkanoates (PHAs). We assessed the potential impact on the estimated aerobic NO_3^- consumption rates in scenario 2 of PHA accumulation in the anoxic phase and its subsequent consumption in the oxic period. Biomass contains 4.2 electrons per carbon, whereas polyhydroxybutyrate (PHB, the most common form of PHA) contains 4.5 electrons per carbon, so changes in the electron balance were minimal. Assuming that 50% of the biomass growth in the anoxic phase was actually PHA accumulation, the estimated aerobic and anaerobic NO_3^- consumption rates were 0.50 ± 0.14 and 1.09 ± 0.53 N-mmol/h (R_4) and 0.20 ± 0.18 and 1.29 ± 0.30 N-mmol/h (R_{32}), nearly identical to scenario 2. Therefore, our conclusions would remain unchanged even in the case of significant PHA accumulation.

Confirmation of the aerobic denitrification rates through concentration profiles. The aerobic denitrification rates obtained in scenario 2 using mass balances, and reported in the

main text, were confirmed with cycle measurements performed after 43-44 days of operation (Figure S8).

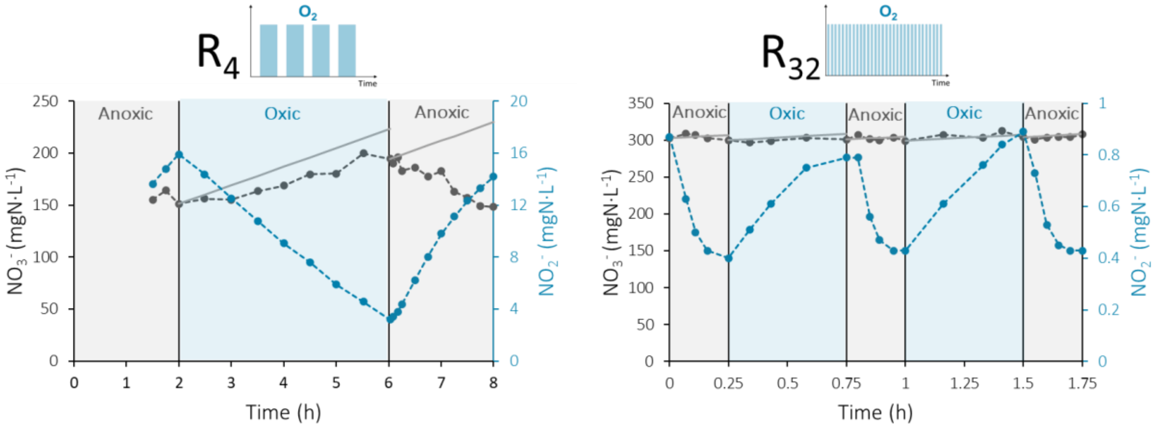

Figure S8. Nitrate and nitrite concentration profiles used to confirm the aerobic denitrification rates. Nitrate concentrations (grey symbols), expected nitrate concentrations if there was no consumption (grey lines), and nitrite concentrations (blue symbols) during the anoxic (grey area) and oxic (blue area) periods are represented. The measurements were performed after 43 (R_4) and 44 (R_{32}) days of operation.

The aerobic nitrate and nitrite net accumulation rates were determined by calculating the slope of the linear regression of the concentration profiles (Figure S8) and multiplying by the broth volume. For R_{32}, the rates measured during two cycles were averaged. The error of these rates was assumed to be the error in the slope (R_4) or the standard deviation of replicates (R_{32}). The nitrate consumption rates were calculated from these accumulation rates and the influent rate (Table S1):

$$R_{NO_3^-,cons} = R_{NO_3^-,accum} - R_{NO_3^-,in} \qquad \text{(eq. S20)}$$

There was no nitrite in the influent but it was continuously produced from nitrate reduction, so the net consumption rates were equal to the accumulation rates added to the nitrate consumption rates ($R_{NO2-,prod} = R_{NO3-,cons}$).

$$R_{NO_2^-,cons} = R_{NO_2^-,accum} - R_{NO_2^-,prod} \qquad \text{(eq. S21)}$$

The obtained aerobic NO_x^- denitrification rates were 0.47±0.07 (R_4) and 0.33±0.16 mmol-N·h^{-1} (R_{32}), similar to the rates obtained through mass balances with eq. S19. The percentage of aerobic electrons used in denitrification vs. O_2 respiration, 33±4% (R_4) and 27±9% (R_{32}), were also similar to the values obtained through mass balances (Figure S9).

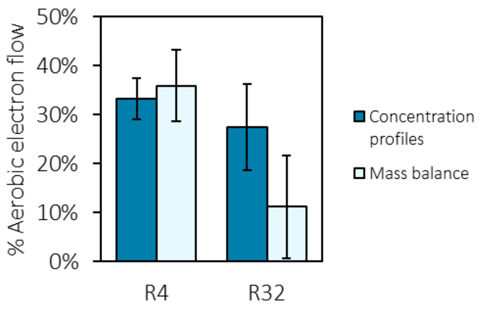

Figure S9. Percentage of aerobic electron flow used in denitrification in both reactors, as calculated through concentration profiles measured on a single day and overall steady-state mass balances (scenario 2 in Figure S6).

The calculated values were determined from relatively small fluctuations in nitrate concentrations, so they were only used to validate the values obtained through the alternative method (mass balances).

3. Nitrification assays

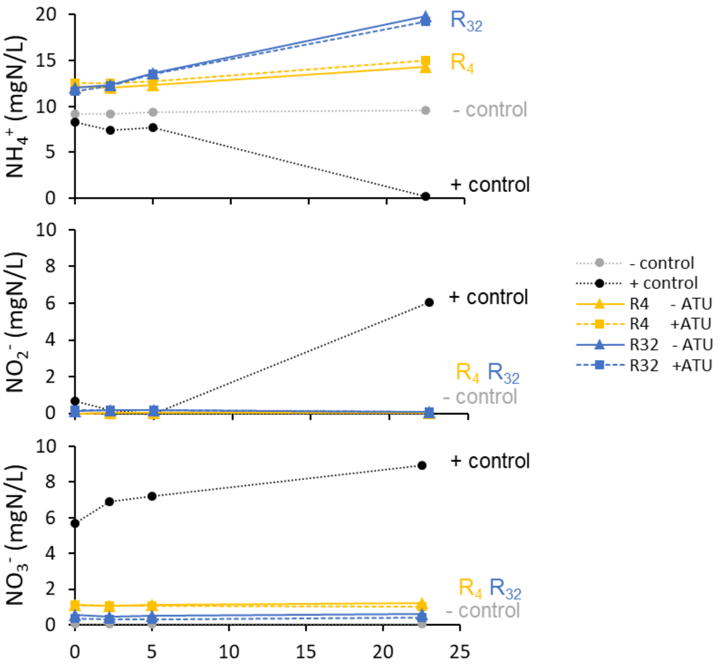

Figure S10. Ammonium, nitrite, and nitrate concentration profiles during ammonium oxidation activity tests with biomass extracted from low- (R_4, yellow) and high-frequency (R_{32}, blue) reactors, alongside a negative (water) and a positive control (nitrifying mixed culture). Batches were performed with 10 mg NH_4^+-N/L, in the presence or absence of ATU.

NH_4^+ oxidation activity tests were performed with biomass extracted from R_4 and R_{32}, in the presence and absence of the NH_4^+ oxidation inhibitor ATU. A negative control replaced biomass with water and a positive control contained biomass from an enriched nitrifying microbial community. The nitrifying culture at pH 7 with a biomass concentration of 0.04 gVSS/L was enriched from activated sludge in a 2 L continuously-stirred tank reactor for 53 days, with an HRT of 4.2 days, using NH_4^+ as energy source (supplied at 34 NH_4^+-N mmol/d), bicarbonate as carbon source, and O_2 as electron acceptor (provided as air at 500 mL/min). The nitrifying biomass was centrifuged and the pellet was resuspended in PBS buffer and added to rubber sealed bottles (filled with air) to prevent excessive evaporation. The bottles were incubated overnight in a shaker at room temperature after addition of 10 mg-N/L NH_4^+ to start the batches. NH_4^+ consumption and NO_2^- and NO_3^- production were observed only in the positive control. NH_4^+ concentrations increased in the experiments with biomass from R_4 and R_{32}, indicating biomass decay. Identical concentration profiles between experiments performed with or without ATU further confirm the absence of NH_4^+ oxidation activity in R_4 and R_{32}.

4. Metagenomics

Table S7. Characteristics of the draft genomes recovered from R_4 ordered from high to low abundance (top 10 + others): Genbank accession number, genome completeness, contamination, and size, GC content, number of predicted genes, relative abundance at 68 days of operation, and taxonomic classification. Bins with a completeness lower than 70% or contamination above 10% were grouped with the unbinned portion. All medium- (MQ) and low-abundant high-quality (HQ) bins were grouped into "others" (grey) in the main manuscript.

	Bin	Genome accession	Comp(%)	Cont(%)	Genome (Mbp)	GC(%)	Genes	Abund(%)	Phylum	Class	Order	Family	Genus	Species
HQ	Bin1.1	JAUCBR000000000	98.96	0.00	5.0	66.1	4695	40.00	*Proteobacteria*	*Gammaproteobacteria*	*Burkholderiales*	*Rhodocyclaceae*	*Denitromonas*	
HQ	Bin1.2	JAUCBS000000000	98.96	0.91	5.5	66.7	5347	17.37	*Proteobacteria*	*Alphaproteobacteria*	*Rhodobacterales*	*Rhodobacteraceae*	*Wagnerdoeblera*	
HQ	Bin1.3	JAUCBT000000000	95.89	1.05	3.8	60.8	3400	7.57	*Proteobacteria*	*Alphaproteobacteria*	*Rhizobiales*	*Xanthobacteraceae*	*Xanthobacter*	
HQ	Bin1.4	JAUCBU000000000	99.61	0.00	2.8	63.9	2582	3.01	*Proteobacteria*	*Gammaproteobacteria*	*Burkholderiales*	*Burkholderiaceae*	*Brachymonas*	*Brachymonas denitrificans*
HQ	Bin1.5	JAUCBV000000000	99.53	2.24	3.9	59.4	3859	2.37	*Proteobacteria*	*Gammaproteobacteria*	*Burkholderiales*	*Burkholderiaceae*	*Castellaniella*	
HQ	Bin1.6	JAUCBW000000000	98.13	0.29	2.9	36.3	2601	2.35	*Bacteroidota*	*Bacteroidia*	*Flavobacteriales*	*Flavobacteriaceae*		
HQ	Bin1.7	JAUCBX000000000	99.44	0.81	4.7	63.2	4678	1.62	*Proteobacteria*	*Alphaproteobacteria*	*Rhodobacterales*	*Rhodobacteraceae*	*Paracoccus*	*Paracoccus sp002359815*
HQ	Bin1.8	JAUCBY000000000	94.12	0.00	3.3	60.5	2965	1.24	*Proteobacteria*	*Alphaproteobacteria*	*Rhizobiales*	*Xanthobacteraceae*	*Xanthobacter*	
HQ	Bin1.9	JAUCBZ000000000	98.48	3.89	4.5	62.5	4466	1.23	*Proteobacteria*	*Alphaproteobacteria*	*Rhodobacterales*	*Rhodobacteraceae*	*Pararhodobacter*	
HQ	Bin1.10	JAUCCA000000000	100	1.67	4.1	61.7	3805	1.20	*Proteobacteria*	*Gammaproteobacteria*	*Burkholderiales*	*Burkholderiaceae*	*Giesbergeria*	
HQ	Bin1.11	JAUCCB000000000	99.51	0.49	2.7	34.5	2528	1.01	*Bacteroidota*	*Bacteroidia*	*Chitinophagales*	*Chitinophagaceae*	*Ginsengibacter*	
HQ	Bin1.12	JAUCCC000000000	97.59	1.09	3.8	48.1	3649	0.57	*Proteobacteria*	*Gammaproteobacteria*	*Pseudomonadales*	*Pseudomonadaceae*	*Pseudomonas_C*	
MQ	Bin1.13	JAUCCD000000000	94.83	6.90	3.7	63.3	3666	0.46	*Proteobacteria*	*Gammaproteobacteria*	*Burkholderiales*	*Rhodocyclaceae*	*Thauera*	
HQ	Bin1.14	JAUCCE000000000	97.05	1.14	4.0	38.9	3659	0.40	*Bacteroidota*	*Bacteroidia*	*Flavobacteriales*	*Flavobacteriaceae*	*Aequorivita*	
HQ	Bin1.15	JAUCCF000000000	90.82	3.82	3.7	64.7	3856	0.26	*Proteobacteria*	*Gammaproteobacteria*	*Burkholderiales*	*Burkholderiaceae*	*Giesbergeria*	
MQ	Bin1.16	JAUCCG000000000	81.01	0.55	2.8	67.2	3490	0.25	*Proteobacteria*	*Alphaproteobacteria*	*Caulobacterales*	*Caulobacteraceae*	*Brevundimonas*	
MQ	Bin1.17	JAUCCH000000000	78.5	1.27	2.9	60.0	3425	0.24	*Actinobacteriota*	*Actinomycetia*	*Actinomycetales*	*Microbacteriaceae*	*Leucobacter*	
MQ	Bin1.18	JAUCCI000000000	75.66	6.85	2.5	66.5	2765	0.23	*Proteobacteria*	*Gammaproteobacteria*	*Burkholderiales*	*Burkholderiaceae*	*Castellaniella*	
HQ	Bin1.19	JAUCCJ000000000	91.89	0.26	4.2	55.2	3800	0.10	*Proteobacteria*	*Gammaproteobacteria*	*Burkholderiales*	*Burkholderiaceae*	*Pusillimonas_B*	*Pusillimonas_B sp013416395*
MQ	Bin1.20	JAUCCK000000000	87.25	2.93	3.2	64.7	3091	0.09	*Proteobacteria*	*Gammaproteobacteria*	*Burkholderiales*	*Burkholderiaceae*	*Giesbergeria*	
HQ	Bin1.21	JAUCCL000000000	99.97	0.16	3.9	67.5	3572	0.08	*Proteobacteria*	*Gammaproteobacteria*	*Burkholderiales*	*Burkholderiaceae*	*Castellaniella*	
MQ	Bin1.22	JAUCCM000000000	81.14	2.96	2.7	64.5	2521	0.08	*Proteobacteria*	*Gammaproteobacteria*	*Burkholderiales*	*Burkholderiaceae*	*Comamonas*	
MQ	Bin1.23	JAUCCN000000000	79.66	0.00	1.3	25.7	1183	0.07	*Patescibacteria*	*JAEDAM01*	*BD1-5*	*UBA6164*	*UBA7396*	
HQ	Bin1.24	JAUCCO000000000	96.16	1.83	3.6	34.1	3183	0.07	*Bacteroidota*	*Bacteroidia*	*NS11-12g*	*UKL13-3*	*UBA6183*	
HQ	Bin1.25	JAUCCP000000000	91.25	1.21	3.6	63.5	3680	0.05	*Proteobacteria*	*Alphaproteobacteria*	*Rhodobacterales*	*Rhodobacteraceae*	*Paracoccus*	*Paracoccus sp002359815*
HQ	Bin1.26	JAUCCQ000000000	94.75	2.21	4.7	68.1	4687	0.05	*Proteobacteria*	*Alphaproteobacteria*	*Rhodobacterales*	*Rhodobacteraceae*	*Pararhodobacter*	
HQ	Bin1.27	JAUCCR000000000	94.12	1.23	3.5	63.9	3394	0.03	*Proteobacteria*	*Gammaproteobacteria*	*Burkholderiales*	*Burkholderiaceae*	*Giesbergeria*	*Giesbergeria suum*
HQ	Bin1.28	JAUCCS000000000	99.17	1.64	4.7	37.9	4055	0.02	*Bacteroidota*	*Bacteroidia*	*Flavobacteriales*	*Flavobacteriaceae*	*Gelidibacter*	*Gelidibacter japonicus*
MQ	Bin1.29	JAUCCT000000000	86.78	0.99	2.7	32.7	2849	0.01	*Bacteroidota*	*Bacteroidia*	*Chitinophagales*	*JADIYW01*		
	Unbinned							17.99						

Table S8. Characteristics of the draft genomes recovered from R_{32} ordered from high to low abundance (top 10 + others): Genbank accession number, genome completeness, contamination, and size, GC content, number of predicted genes, relative abundance at 68 days of operation, and taxonomic classification. Bins with a completeness lower than 70% or contamination above 10% were grouped with the unbinned portion. All medium- (MQ) and low-abundant high-quality (HQ) bins were grouped into "others" (grey) in the main manuscript.

	Bin	Genome accession	Comp(%)	Cont(%)	Genome (Mbp)	GC(%)	Genes	Abund(%)	Phylum	Class	Order	Family	Genus	Species
HQ	Bin2.1	JAUCCU000000000	98.85	0.00	3.2	59.3	3036	23.52	*Proteobacteria*	*Gammaproteobacteria*	*Burkholderiales*	*Burkholderiaceae*	*Castellaniella*	
HQ	Bin2.2	JAUCCV000000000	99.37	2.40	3.1	62.2	2902	10.61	*Proteobacteria*	*Gammaproteobacteria*	*Burkholderiales*	*Burkholderiaceae*	*Castellaniella*	
HQ	Bin2.3	JAUCCW000000000	98.81	1.76	4.3	63.0	3964	7.32	*Proteobacteria*	*Gammaproteobacteria*	*Burkholderiales*	*Rhodocyclaceae*	*Thauera*	
HQ	Bin2.4	JAUCCX000000000	98.29	0.40	3.6	36.9	3206	5.02	*Bacteroidota*	*Bacteroidia*	*Flavobacteriales*	*Flavobacteriaceae*	*Aequorivita*	
HQ	Bin2.5	JAUCCY000000000	99.41	0.47	3.9	68.9	3479	2.67	*Proteobacteria*	*Gammaproteobacteria*	*Burkholderiales*	*Burkholderiaceae*	*Castellaniella*	*Castellaniella defragrans*
HQ	Bin2.6	JAUCCZ000000000	93.95	2.25	3.1	61.8	2958	2.13	*Proteobacteria*	*Gammaproteobacteria*	*Burkholderiales*	*Burkholderiaceae*	*Comamonas*	
HQ	Bin2.7	JAUCDA000000000	98.99	0.91	3.8	59.2	3671	1.65	*Proteobacteria*	*Alphaproteobacteria*	*Rhodobacterales*	*Rhodobacteraceae*	*Pseudorhodobacter*	
HQ	Bin2.8	JAUCDB000000000	97.73	3.66	5.0	63.0	5008	1.52	*Proteobacteria*	*Alphaproteobacteria*	*Rhodobacterales*	*Rhodobacteraceae*	*Paracoccus*	*Paracoccus sp002359815*
HQ	Bin2.9	JAUCDC000000000	96.7	2.46	3.0	37.2	2922	1.44	*Bacteroidota*	*Bacteroidia*	*Flavobacteriales*	*Flavobacteriaceae*		
HQ	Bin2.10	JAUCDD000000000	96.36	4.9	3.9	68.8	3558	0.95	*Proteobacteria*	*Gammaproteobacteria*	*Burkholderiales*	*Burkholderiaceae*	*Melaminivora_A*	
MQ	Bin2.11	JAUCDE000000000	80.22	0.00	1.3	26.6	1250	6.53	*Patescibacteria*	*JAEDAM01*	*BD1-5*	*UBA6164*	*UBA7396*	*UBA7396 sp002470645*
MQ	Bin2.12	JAUCDF000000000	94.69	7.47	3.8	62.7	3530	3.76	*Proteobacteria*	*Gammaproteobacteria*	*Burkholderiales*	*Burkholderiaceae*	*Comamonas*	*Comamonas sp019104825*
MQ	Bin2.13	JAUCDG000000000	97.7	7.04	4.1	48.4	3929	2.18	*Proteobacteria*	*Gammaproteobacteria*	*Pseudomonadales*	*Pseudomonadaceae*	*Pseudomonas_C*	
MQ	Bin2.14	JAUCDH000000000	88.51	2.61	3.1	65.9	3002	0.95	*Proteobacteria*	*Gammaproteobacteria*	*Xanthomonadales*	*Xanthomonadaceae*	*Stenotrophomonas*	
MQ	Bin2.15	JAUCDI000000000	77.08	1.63	1.8	43.1	1964	0.89	*Proteobacteria*	*Alphaproteobacteria*	*Paracaedibacterales*	*Paracaedibacteraceae*		
MQ	Bin2.16	JAUCDJ000000000	92.88	5.36	3.7	64.8	3865	0.81	*Proteobacteria*	*Gammaproteobacteria*	*Burkholderiales*	*Burkholderiaceae*	*Giesbergeria*	
MQ	Bin2.17	JAUCDK000000000	81.35	0.00	1.2	25.9	1111	0.35	*Patescibacteria*	*JAEDAM01*	*BD1-5*	*UBA6164*	*UBA7396*	
HQ	Bin2.18	JAUCDL000000000	98	0.03	3.3	68.8	3065	0.33	*Proteobacteria*	*Gammaproteobacteria*	*Burkholderiales*	*Burkholderiaceae*	*Castellaniella*	
HQ	Bin2.19	JAUCDM000000000	90.25	3.15	3.1	64.1	2759	0.27	*Proteobacteria*	*Gammaproteobacteria*	*Burkholderiales*	*Burkholderiaceae*	*Comamonas*	
HQ	Bin2.20	JAUCDN000000000	92.18	2.05	3.5	64.6	3616	0.20	*Proteobacteria*	*Alphaproteobacteria*	*Rhizobiales*	*Devosiaceae*	*Devosia*	
HQ	Bin2.21	JAUCDO000000000	97.98	1.84	3.9	38.7	3378	0.16	*Bacteroidota*	*Bacteroidia*	*Flavobacteriales*	*Flavobacteriaceae*	*Aequorivita*	
HQ	Bin2.22	JAUCDP000000000	97.27	3.95	4.5	62.3	4359	0.10	*Proteobacteria*	*Gammaproteobacteria*	*Burkholderiales*	*Rhodocyclaceae*	*Azoarcus_C*	
HQ	Bin2.23	JAUCDQ000000000	93.02	1.94	3.8	60.2	3838	0.05	*Proteobacteria*	*Alphaproteobacteria*	*Rhizobiales*	*Rhizobiaceae*	*Hoeflea*	
MQ	Bin2.24	JAUCDR000000000	78.42	3.71	3.2	67.1	3605	0.04	*Proteobacteria*	*Alphaproteobacteria*	*Sphingomonadales*	*Sphingomonadaceae*	*Sphingopyxis*	*Sphingopyxis granuli*
HQ	Bin2.25	JAUCDS000000000	99.29	0.5	3.2	64.5	3015	0.04	*Proteobacteria*	*Gammaproteobacteria*	*Burkholderiales*	*Burkholderiaceae*	*Castellaniella*	
HQ	Bin2.26	JAUCDT000000000	92.51	1.00	3.1	68.1	2884	0.04	*Proteobacteria*	*Gammaproteobacteria*	*Burkholderiales*	*Burkholderiaceae*	*Comamonas_C*	*Comamonas_C sp002894305*
MQ	Bin2.27	JAUCDU000000000	77.62	2.16	2.6	64.0	2734	0.04	*Proteobacteria*	*Gammaproteobacteria*	*Burkholderiales*	*Burkholderiaceae*	*Giesbergeria*	
MQ	Bin2.28	JAUCDV000000000	77.69	0.98	2.0	61.5	2259	0.03	*Actinobacteriota*	*Actinomycetia*	*Actinomycetales*	*Microbacteriaceae*	*Leucobacter*	
MQ	Bin2.29	JAUCDW000000000	88.65	2.51	2.9	48.0	3153	0.02	*Proteobacteria*	*Gammaproteobacteria*	*Pseudomonadales*	*Pseudomonadaceae*	*Pseudomonas_C*	
	Unbinned							26.38						

Table S9. Reference KO-numbers of the genes from the nitrogen metabolism.

KO ID	Gene	Description	Pathway
K00362	nirB	nitrite reductase (NADH) large subunit [EC:1.7.1.15]	
K00363	nirD	nitrite reductase (NADH) small subunit [EC:1.7.1.15]	DNRA
K03385	nrfA	nitrite reductase (cytochrome c-552) [EC:1.7.2.2]	
K15876	nrfH	cytochrome c nitrite reductase small subunit	
K00370	narG, narZ, nxrA	nitrate reductase / nitrite oxidoreductase, alpha subunit [EC:1.7.5.1 1.7.99.-]	
K00371	narH, narY, nxrB	nitrate reductase / nitrite oxidoreductase, beta subunit [EC:1.7.5.1 1.7.99.-]	
K00373	narJ, narW	chaperone	
K00374	narI, narV	nitrate reductase gamma subunit [EC:1.7.5.1 1.7.99.-]	
K07673	narX	nitrate/nitrite sensor	
K07684	narL	response regulator	
K02575	NRT, narK, nrtP, nasA	MFS transporter, NNP family, nitrate/nitrite transporter	
K02567	napA	nitrate reductase (cytochrome) [EC:1.9.6.1]	
K02568	napB	nitrate reductase (cytochrome), electron transfer subunit	
K02570	napD	nitrate reductase (cytochrome)	
K02571	napE	nitrate reductase (cytochrome)	
K00367	narB	ferredoxin-nitrate reductase	
K10850	narT	putative nitrate transporter	
K15576	nrtA, nasF, cynA	nitrate/nitrite transport system substrate-binding protein	
K15577	nrtB, nasE, cynB	nitrate/nitrite transport system permease protein	
K15578	nrtC, nasD	nitrate/nitrite transport system ATP-binding protein [EC:7.3.2.4]	
K15579	nrtD, cynD	nitrate/nitrite transport system ATP-binding protein	
K21563	dnr	CRP/FNR family transcriptional regulator, dissimilatory nitrate respiration regulator	Denitrification
K01420	fnr	CRP/FNR family transcriptional regulator, anaerobic regulatory protein	
K00368	nirK	nitrite reductase (NO-forming) [EC:1.7.2.1]	
K15864	nirS	nitrite reductase (NO-forming) / hydroxylamine reductase [EC:1.7.2.1 1.7.99.1]	
K04561	norB	nitric oxide reductase subunit B [EC:1.7.2.5]	
K02305	norC	nitric oxide reductase subunit C	
KnorZ	norZ	quinol-dependent nitric oxide reductase	
K02448	norD	nitric oxide reductase D protein	
K02164	norE	nitric oxide reductase E protein	
K04747	norF	nitric oxide reductase F protein	
K04748	norQ	nitric oxide reductase Q protein	
K12266	treg	transcription regulator	
K13771	trep	NO-sensitive transcription repressor	
K00376	nosZ I	nitrous-oxide reductase [EC:1.7.2.4]	
KnosZII	nosZ II	nitrous-oxide reductase [EC:1.7.2.4]	
K19339	nosR	nitrous-oxide reductase transcriptional regulator	
K19342	nosL	copper chaperone	
K07218	nosD	accessory protein	
K10944	amoA	methane/ammonia monooxygenase subunit A [EC:1.14.18.3 1.14.99.39]	
K10945	amoB	methane/ammonia monooxygenase subunit B	Nitrification
K10946	amoC	methane/ammonia monooxygenase subunit C	
K10535	hao	hydroxylamine dehydrogenase [EC:1.7.2.6]	

Table S10. Reference KO-numbers of the genes from the respiratory chain.

KO ID	Gene	Description	Reaction type
K00330	nuoA	NADH-quinone oxidoreductase subunit A [EC:7.1.1.2]	
K00331	nuoB	NADH-quinone oxidoreductase subunit B [EC:7.1.1.2]	
K00332	nuoC	NADH-quinone oxidoreductase subunit C [EC:7.1.1.2]	
K00333	nuoD	NADH-quinone oxidoreductase subunit D [EC:7.1.1.2]	
K00334	nuoE	NADH-quinone oxidoreductase subunit E [EC:7.1.1.2]	
K00335	nuoF	NADH-quinone oxidoreductase subunit F [EC:7.1.1.2]	
K00336	nuoG	NADH-quinone oxidoreductase subunit G [EC:7.1.1.2]	
K00337	nuoH	NADH-quinone oxidoreductase subunit H [EC:7.1.1.2]	NADH oxidation
K00338	nuoI	NADH-quinone oxidoreductase subunit I [EC:7.1.1.2]	
K00339	nuoJ	NADH-quinone oxidoreductase subunit J [EC:7.1.1.2]	
K00340	nuoK	NADH-quinone oxidoreductase subunit K [EC:7.1.1.2]	
K00341	nuoL	NADH-quinone oxidoreductase subunit L [EC:7.1.1.2]	
K00342	nuoM	NADH-quinone oxidoreductase subunit M [EC:7.1.1.2]	
K00343	nuoN	NADH-quinone oxidoreductase subunit N [EC:7.1.1.2]	
K03885	ndh	NADH:quinone reductase (non-electrogenic) [EC:1.6.5.9]	
K00411	petA	ubiquinol-cytochrome c reductase iron-sulfur subunit [EC:7.1.1.8]	
K00412	petB	ubiquinol-cytochrome c reductase cytochrome b subunit	
K00413	petC	ubiquinol-cytochrome c reductase cytochrome c1 subunit	
K00410	fbcH	ubiquinol-cytochrome c reductase cytochrome b/c1 subunit	Cyt c reduction
K03890	qcrA	ubiquinol-cytochrome c reductase iron-sulfur subunit	
K03891	qcrB	ubiquinol-cytochrome c reductase cytochrome b subunit	
K03889	qcrC	ubiquinol-cytochrome c reductase cytochrome c subunit	
K02274	coxA,ctaD	cytochrome *aa*3 oxidase subunit I [EC:7.1.1.9]	
K02275	coxB,ctaC	cytochrome *aa*3 oxidase subunit II [EC:7.1.1.9]	
K02276	coxC,ctaE	cytochrome *aa*3 oxidase subunit III [EC:7.1.1.9]	
K02277	coxD,ctaF	cytochrome *aa*3 oxidase subunit IV [EC:7.1.1.9]	
K00404	ccoN	cytochrome *cbb*3 oxidase subunit I [EC:7.1.1.9]	
K00405	ccoO	cytochrome *cbb*3 oxidase subunit II	
K15862	ccoNO	cytochrome *cbb*3 oxidase subunit I/II [EC:7.1.1.9]	
K00407	ccoQ	cytochrome *cbb*3 oxidase subunit IV	
K00406	ccoP	cytochrome *cbb*3 oxidase subunit III	O_2 reduction
K02297	cyoA	cytochrome *bo*3 ubiquinol oxidase subunit I [EC:7.1.1.3]	
K02298	cyoB	cytochrome *bo*3 ubiquinol oxidase subunit II [EC:7.1.1.3]	
K02299	cyoC	cytochrome *bo*3 ubiquinol oxidase subunit III	
K02300	cyoD	cytochrome *bo*3 ubiquinol oxidase subunit IV	
K00425	cydA	cytochrome *bd* ubiquinol oxidase subunit I [EC:7.1.1.7]	
K00426	cydB	cytochrome *bd* ubiquinol oxidase subunit II [EC:7.1.1.7]	
K00424	cydX	cytochrome *bd* ubiquinol oxidase subunit X [EC:7.1.1.7]	
K08738	CYC	cytochrome c	Cytochrome c
K02111	atpA	F-type H+/Na+-transporting ATPase subunit alpha [EC:7.1.2.2 7.2.2.1]	
K02108	atpB	F-type H+-transporting ATPase subunit a	
K02114	atpC	F-type H+-transporting ATPase subunit epsilon	
K02112	atpD	F-type H+/Na+-transporting ATPase subunit beta [EC:7.1.2.2 7.2.2.1]	
K02110	atpE	F-type H+-transporting ATPase subunit c	ATP synthesis
K02109	atpF	F-type H+-transporting ATPase subunit b	
K02115	atpG	F-type H+-transporting ATPase subunit gamma	
K02113	atpH	F-type H+-transporting ATPase subunit delta	

Table S11. Reference KO-numbers of the genes from the ROS-protection pathway.

KO ID	Gene	Description	Reaction type
K04565	SOD1	superoxide dismutase, Cu-Zn family [EC:1.15.1.1]	$O_2^- \rightarrow H_2O_2$
K04564	SOD2	superoxide dismutase, Fe-Mn family [EC:1.15.1.1]	
K03781	katE, catB, srpA	catalase [EC:1.11.1.6]	
K07217	Mn-cat	Mn-catalase	
K03782	katG	catalase-peroxidase [EC:1.11.1.21]	
K00428	ccp	cytochrome c peroxidase [EC:1.11.1.5]	
K00430	px	peroxidase	
K11065	tpx	thiol peroxidase	$H_2O_2 \rightarrow H_2O$
K00432	gpx, btuE, bsaA	glutathione peroxidase	
K05910	npr	NADH peroxidase	
K14171	ahp1	alkyl hydroperoxide reductase	
K03386	ahpC	alkyl hydroperoxide reductase	

5. Heatmaps with gene presence and protein expression

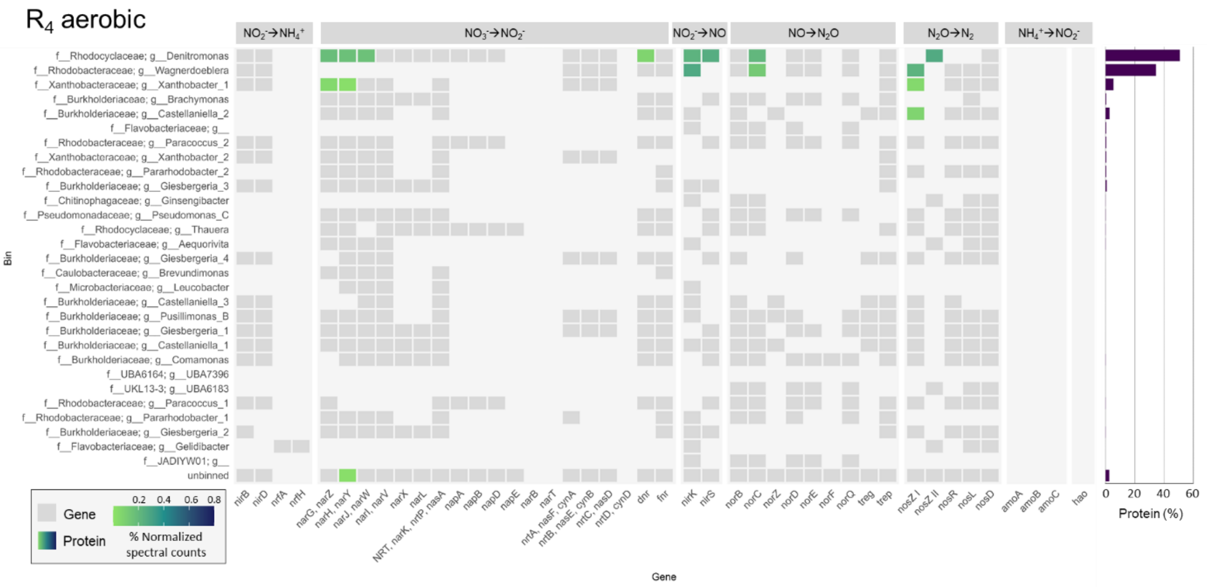

Figure S11. Heatmap with gene presence (grey) and protein expression (coloured) of the nitrogen metabolism, represented as relative abundance of the total proteome, of all MAGs (ordered from high to low abundance in the metagenome) at the end of the oxic phase of R_4. **Right bar charts:** total relative abundance of each MAG in the metaproteome.

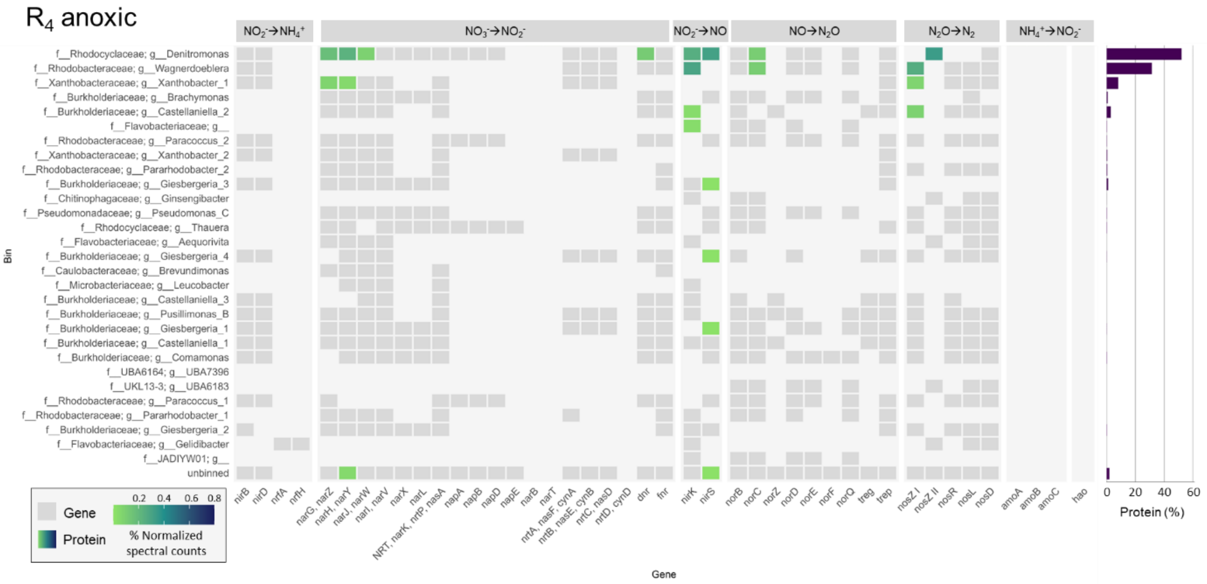

Figure S12. Heatmap with gene presence (grey) and protein expression (coloured) of the nitrogen metabolism, represented as relative abundance of the total proteome, of all MAGs (ordered from high to low abundance in the metagenome) at the end of the anoxic phase of R_4. **Right bar charts:** total relative abundance of each MAG in the metaproteome.

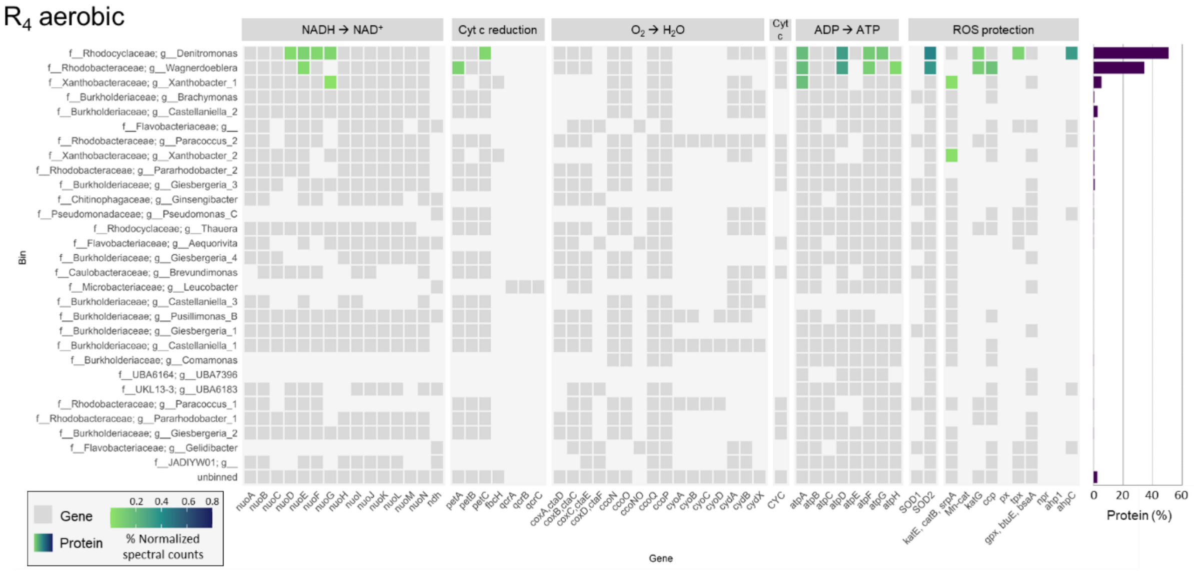

Figure S13. Heatmap with gene presence (grey) and protein expression (coloured) of the respiratory chain and ROS-protection pathway, represented as relative abundance of the total proteome, of all MAGs (ordered from high to low abundance in the metagenome) at the end of the oxic phase of R_4. **Right bar charts:** total relative abundance of each MAG in the metaproteome.

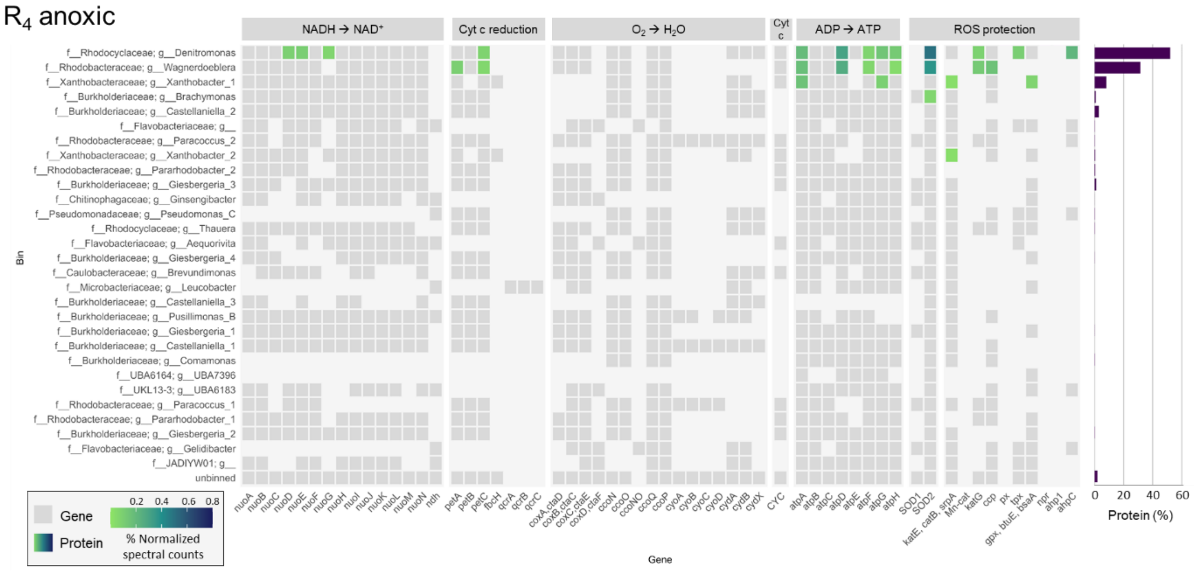

Figure S14. Heatmap with gene presence (grey) and protein expression (coloured) of respiratory chain and ROS-protection pathway, represented as relative abundance of the total proteome, of all MAGs (ordered from high to low abundance in the metagenome) at the end of the anoxic phase of R_4. **Right bar charts:** total relative abundance of each MAG in the metaproteome.

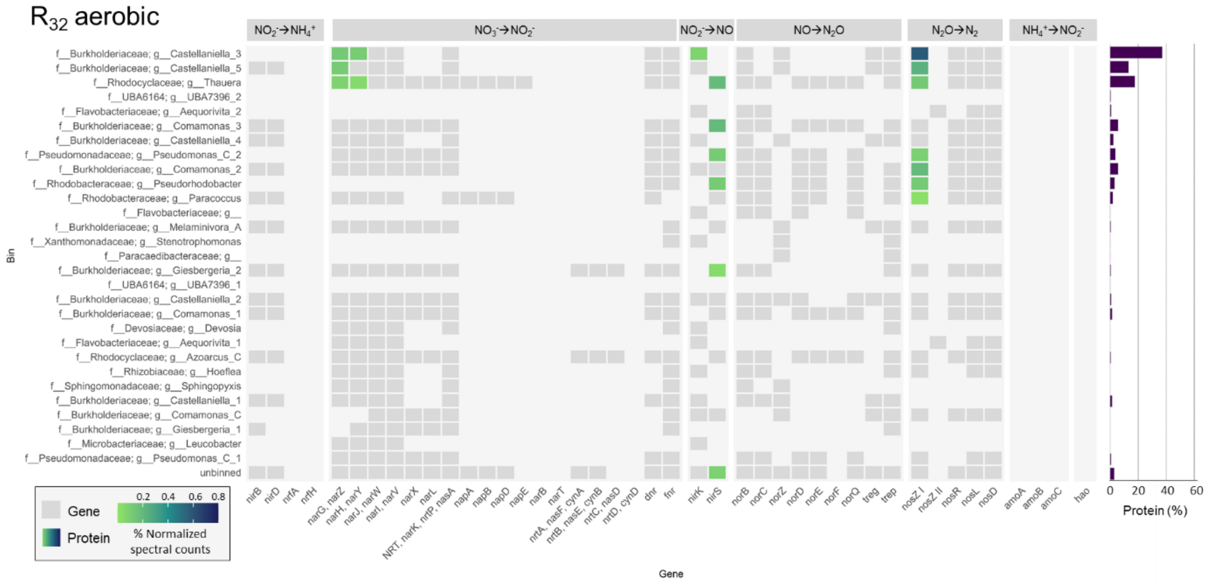

Figure S15. Heatmap with gene presence (grey) and protein expression (coloured) of the nitrogen metabolism, represented as relative abundance of the total proteome, of all MAGs (ordered from high to low abundance in the metagenome) at the end of the oxic phase of R_{32}. **Right bar charts:** total relative abundance of each MAG in the metaproteome.

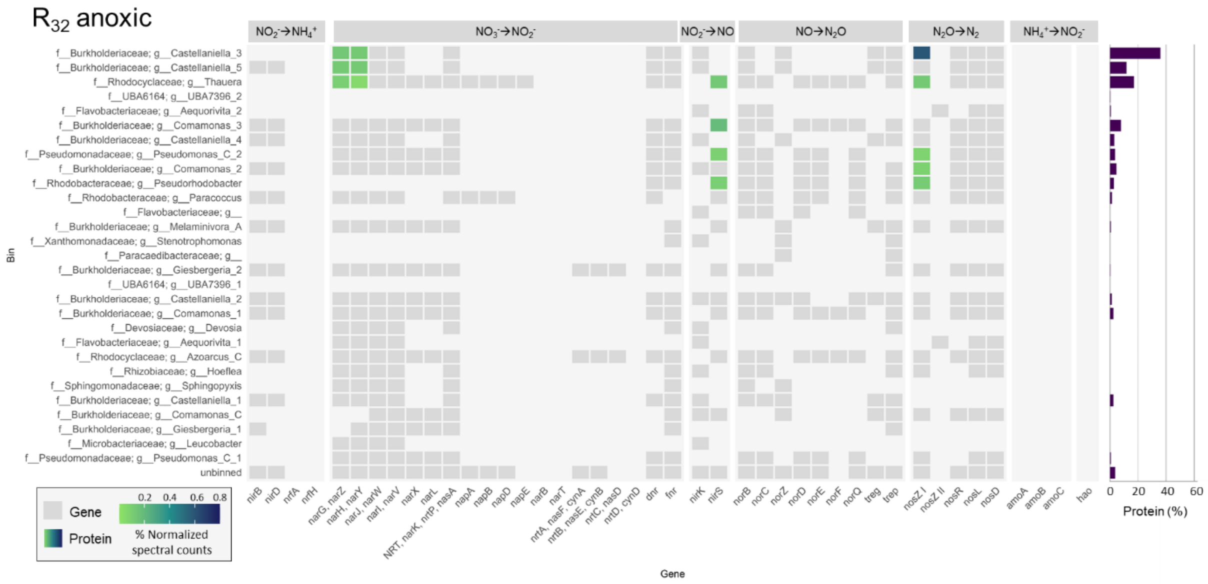

Figure S16. Heatmap with gene presence (grey) and protein expression (coloured) of the nitrogen metabolism, represented as relative abundance of the total proteome, of all MAGs (ordered from high to low abundance in the metagenome) at the end of the anoxic phase of R_{32}. **Right bar charts:** total relative abundance of each MAG in the metaproteome.

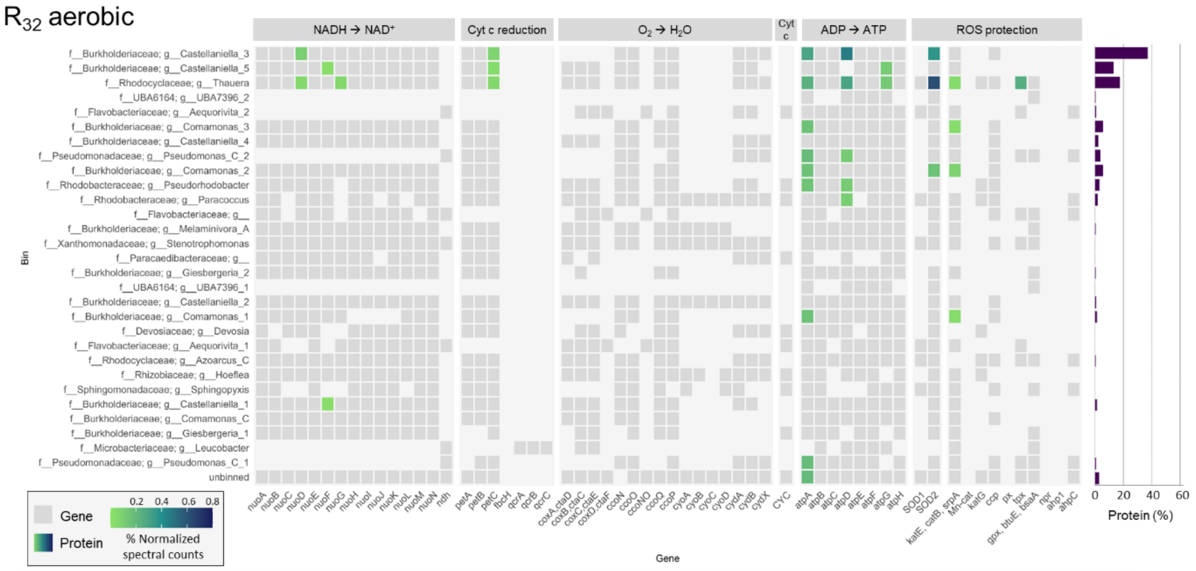

Figure S17. Heatmap with gene presence (grey) and protein expression (coloured) of respiratory chain and ROS-protection pathway, represented as relative abundance of the total proteome, of all MAGs (ordered from high to low abundance in the metagenome) at the end of the oxic phase of R_{32}. **Right bar charts:** total relative abundance of each MAG in the metaproteome.

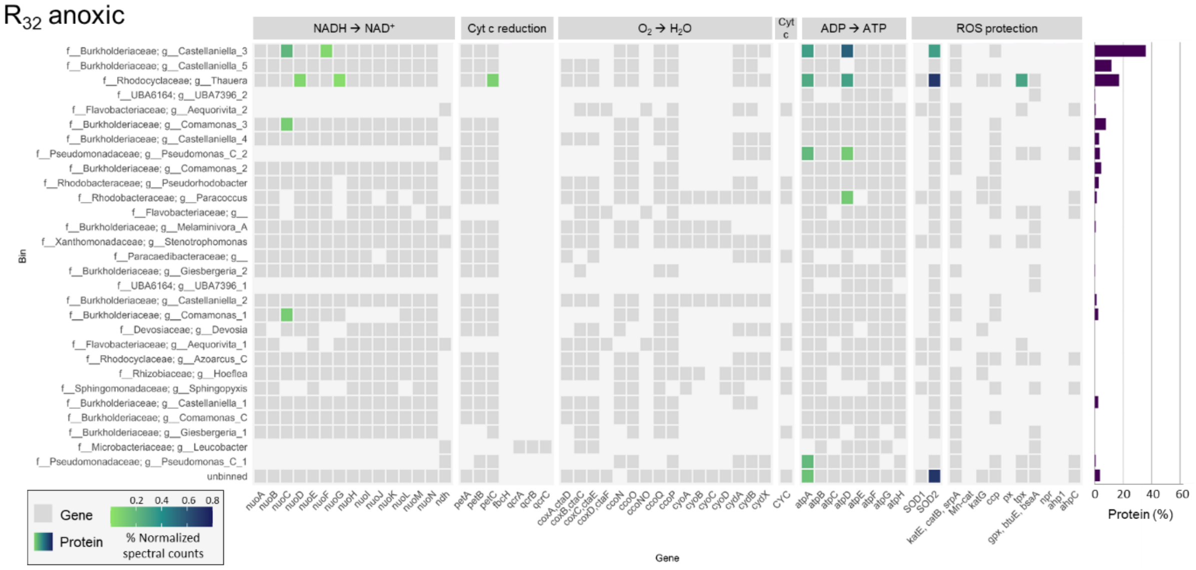

Figure S18. Heatmap with gene presence (grey) and protein expression (coloured) of respiratory chain and ROS-protection pathway, represented as relative abundance of the total proteome, of all MAGs (ordered from high to low abundance in the metagenome) at the end of the anoxic phase of R_{32}. **Right bar charts:** total relative abundance of each MAG in the metaproteome.

6. Oxic/anoxic cycling in 5 reference Dutch WWTPs

The exposure frequency of activated sludge to oxic/anoxic cycles in wastewater treatment plants (WWTPs) cannot exactly be determined, but estimations were made for different WWTPs using flow rates and tank volumes. The hydraulic residence time in each of the tanks was determined (Figure S19): anaerobic (no O_2, no NO_x), anoxic (no O_2), facultative (can function as anoxic or aerobic tank, according to the treatment needs), and aerobic (with O_2). The sludge residence time in the anoxic tanks varied between 11 and 142 minutes, whereas this was 13-155 min for the aerobic zones. The biomass that passes through the settler experiences approximately one oxic/anoxic transition per day, which is equivalent to 15-42 transitions per sludge retention time (SRT, equivalent to the cell generation time, normally 15-20 days in a WWTP). If cells remain in a recycling loop between the aerobic and anoxic zones they can experience up to 9-36 transitions per day, i.e. 132-756 switches per SRT (Table S12). Similarly to the activated sludge in the WWTPs, the biomass in our reactors experienced 4 (R_4) and 32 (R_{32}) oxic/anoxic transitions per day, equalling 8 and 64 transitions within one SRT. Experiments with even higher frequency of oxic/anoxic transitions within one SRT should be performed to assess the extent of aerobic denitrification in the highest frequency ranges observed in WWTPs.

Table S12. Number of oxic/anoxic transitions experienced by the biomass in our reactors and in five different WWTP configurations (Figure S19), in one day and within one sludge retention time (SRT).

System	SRT (d)	Cycles/day	Cycles/SRT
R_4	2	4	8
R_{32}	2	32	64
WWTP A	15	1 - 35	15 - 315
WWTP B	21	2 - 36	42 - 756
WWTP C	22	1 - 19	22 - 418
WWTP D	15	1 - 14	15 - 210
WWTP E	15	1 - 9	15 - 135

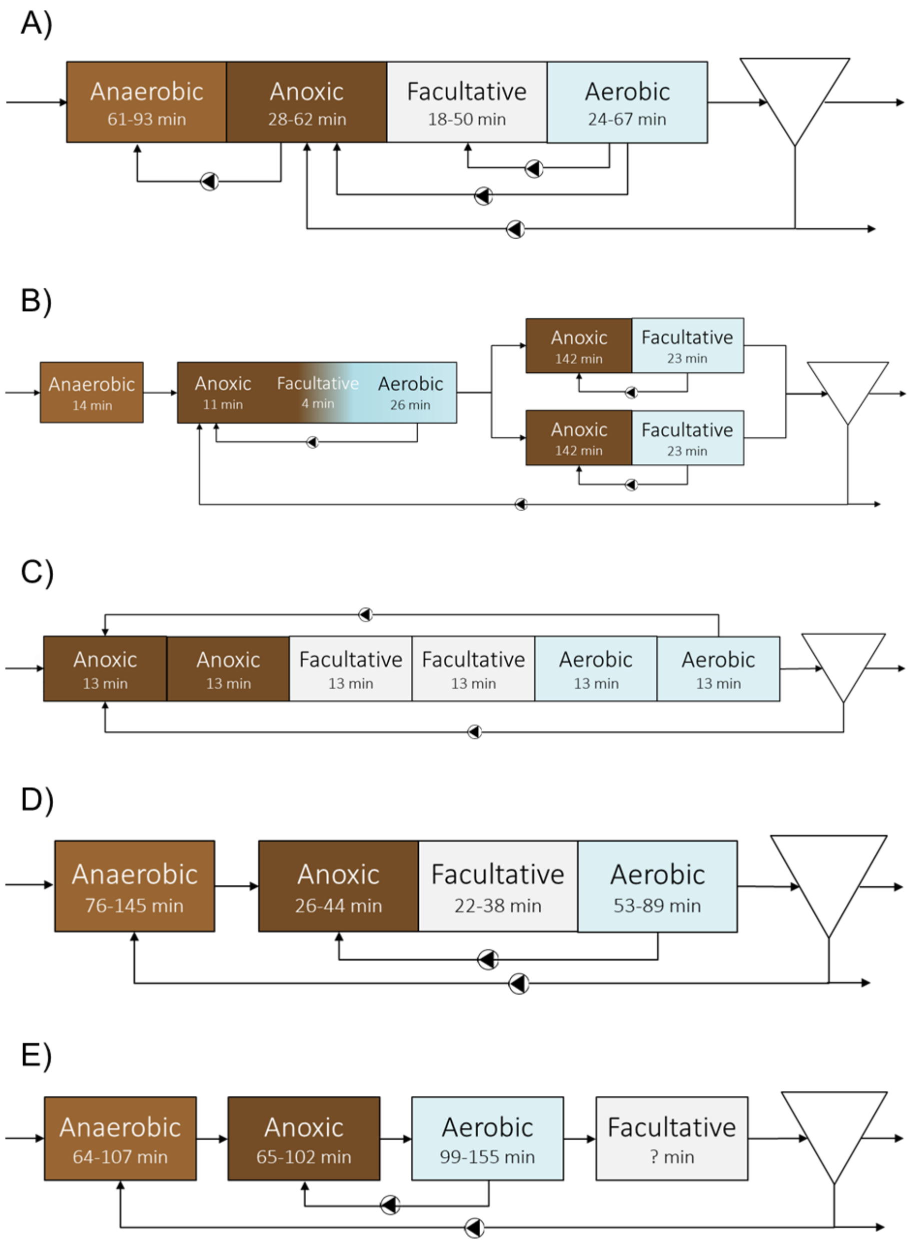

Figure S19. Hydraulic residence time in tanks with different conditions in five different Dutch WWTPs, representing the time that the sludge experiences those conditions. The anaerobic tanks do not contain O_2 nor nitrogen oxides, the anoxic tanks have no oxygen, the aerobic tanks are aerated with air and the facultative tanks can function either as anoxic or aerobic tanks.

7. Microbial composition of the inoculum

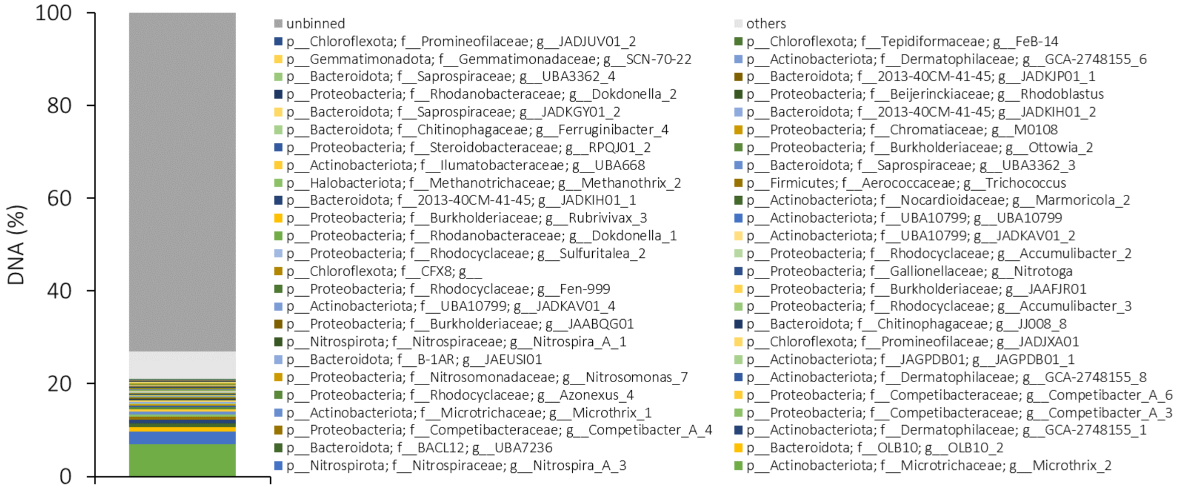

Figure S20. DNA composition of the inoculum (activated sludge). 349 high-quality metagenome-assembled genomes were identified, 305 of these had at least one denitrification gene [1]. The most abundant 50 MAGs are represented here. The activated sludge sample from the full-scale treatment plant was taken two weeks after the inoculation.

1. Roothans N, Pabst M, van Diemen M et al. Long-term multi-meta-omics resolves the ecophysiological controls of seasonal N2O emissions. *bioRxiv* 2024. https://doi.org/10.1101/2024.04.17.589950.
